# Structural stigma and its impact on healthcare for borderline personality disorder: a scoping review

**DOI:** 10.1186/s13033-022-00558-3

**Published:** 2022-09-29

**Authors:** Pauline Klein, A. Kate Fairweather, Sharon Lawn

**Affiliations:** 1 Discipline of Population Health, College of Medicine and Public Health, Flinders University, Adelaide, SA 5001 Australia; 2 Discipline of Behavioural Health, College of Medicine and Public Health, Flinders University, Adelaide, SA 5001 Australia

**Keywords:** Borderline personality disorder, Structural stigma, Health systems, Health services, Healthcare, Crisis care, Health practitioners, Consumers, Carers, Families

## Abstract

**Background:**

People with Borderline Personality Disorder (BPD) and their carers/families continue to experience structural stigma when accessing health services. Structural stigma involves societal-level conditions, cultural norms, and organizational policies that inhibit the opportunities, resources, and wellbeing of people living with attributes that are the object of stigma. BPD is a serious mental illness characterized by pervasive psychosocial dysfunction including, problems regulating emotions and suicidality. This scoping review aimed to identify, map, and explore the international literature on structural stigma associated with BPD and its impact on healthcare for consumers with BPD, their carers/families, and health practitioners.

**Methods:**

A comprehensive search of the literature encompassed MEDLINE, CINAHL, PsycINFO, Scopus, Cochrane Library, and JBI Evidence-Based databases (from inception to February 28th 2022). The search strategy also included grey literature searches and handsearching the references of included studies. Eligibility criteria included citations relevant to structural stigma associated with BPD and health and crisis care services. Quality appraisal of included citations were completed using the Mixed Methods Appraisal Tool 2018 version (MMAT v.18), the Joanna Briggs Institute (JBI) Checklist for Systematic Reviews and Research Syntheses Tool, and the AGREE II: advancing guideline development, reporting, and evaluation in health care tool. Thematic Analysis was used to inform data extraction, analysis, interpretation, and synthesis of the data.

**Results:**

A total of 57 citations were included in the review comprising empirical peer-reviewed articles (n = 55), and reports (n = 2). Studies included quantitative, qualitative, mixed methods, and systematic review designs. Review findings identified several extant macro- and micro-level structural mechanisms, challenges, and barriers contributing to BPD-related stigma in health systems. These structural factors have a substantial impact on health service access and care for BPD. Key themes that emerged from the data comprised: structural stigma and the BPD diagnosis and BPD-related stigma surrounding health and crisis care services.

**Conclusion:**

Narrative synthesis of the findings provide evidence about the impact of structural stigma on healthcare for BPD. It is anticipated that results of this review will inform future research, policy, and practice to address BPD-related stigma in health systems, as well as approaches for improving the delivery of responsive health services and care for consumers with BPD and their carers/families.

*Review Registration*: Open Science Framework (https://osf.io/bhpg4).

**Supplementary Information:**

The online version contains supplementary material available at 10.1186/s13033-022-00558-3.

## Background

Consumers with a diagnosis of BPD and their carers/families are often confronted with structural stigma when accessing health services for their mental health condition [1–4]. Structural stigma is defined as “the societal-level conditions, cultural norms, and institutional policies that constrain the opportunities, resources, and wellbeing of the stigmatized” ([5] p.742). Stigma is a multi-level phenomenon that occurs within various interpersonal, organisational, and structural contexts causing health inequities in accessing services and supports [5], and poor health outcomes [6] for consumers with BPD [3, 7–9] and their carers/families [1, 2, 10, 11]. BPD is a serious mental illness associated with longstanding and persistent patterns of instability in psychosocial functioning, including problems regulating emotions, self-image, interpersonal relationships, impulsivity, and suicidality [12]. The global lifetime prevalence of BPD is approximately 1–2% in the general population [13–16]; affecting 10% of consumers in outpatient settings, and up to 22% of consumers in inpatient settings [13, 17, 18].

BPD is a complex and contentious diagnosis [19], partly because evidence is yet to determine the exact cause of the condition. However, the trajectory is likely to be linked to genetic and environmental factors including emotional vulnerability, childhood abuse [20, 21], and insecure attachment. Some people with BPD may have experienced traumatic childhood events which can impact their ability to form healthy trusting relationships and develop the resilience needed to cope with the pressures of everyday life [22]. While trauma is not necessarily implicated in the suicidality (i.e., self-harm, suicidal ideation, and suicide attempts) common in BPD [12], people with BPD are a high-risk group for suicide [23] which is often triggered by heightened emotions and repetitive cycles of intense distress and crises [24, 25]. Chesney et al. [26] conducted a meta-review on the risk for suicide mortality associated with major psychiatric disorders and found that the suicide risk among consumers with BPD was 45% greater than the general population, and disproportionately higher than other psychiatric disorders. Other studies investigating the prevalence of suicidality found that 75% of people with BPD attempted suicide [27], and up to 10% of people with BPD died by suicide [28].

Recurrent presentations to health services resulting from suicidality among this population place increased demand on health systems [27, 29]. This is particularly evident in emergency services; however, the care provided is often not adequate for meeting the complex needs of consumers with BPD [10]. A recent study investigating the prevalence of mental health presentations reported that consumers with personality disorders represented 20.5% of emergency service presentations and 26.6% of inpatient admissions. Further, consumers with personality disorders were 50% more likely to access health services while experiencing crisis within 28 days of their last presentation, relative to consumers with other mental health disorders [30]. Another study investigating health service utilization found that specialist psychotherapy services, day treatments, residential programs, outpatient, and inpatient medical services were accessed at higher rates by consumers with BPD, than by other consumers [29]. Findings from a community sample also found that 75% of people with BPD accessed help from a range of health professionals including physicians, therapists, and counsellors for their mental illness which may reflect the co-occurring disorders and complexity associated with BPD [31]. The high prevalence of chronic suicidality and crisis presentations to health services by consumers with BPD [15, 27, 28] has contributed to this disorder becoming one of the most highly stigmatized and marginalized mental health conditions in health systems [32, 33].

There is a growing body of research exploring the experiences of BPD-related stigma among consumers with BPD [3, 7–9, 34, 35] and their carers/families [1, 2, 11] when accessing health services. Consumers with BPD consistently report receiving suboptimal levels of care from health services including not being believed or dismissed in relation to the nature and severity of their presentation [3]. These experiences appear to stem from various misconceptions surrounding BPD and suicidality [36]. There are also reports of interactions of conflict between consumers with BPD, their carers/families, and health practitioners [37]. In some instances, consumers with BPD report that they are refused treatment when presenting to health services in distress [38, 39]. Carers/families of consumers with BPD report experiencing anxiety and grief associated with caring for their family member with BPD. Carers/families also experience substantial ongoing financial burdens [11, 40] associated with the costs of private health services including evidence-based therapies and hospitalization of the person with BPD for whom they provide care. Access to clinical and community-based services and supports for consumers with BPD and their carers/families are limited, with current services and supports not adequately meeting the demand for treatment of BPD [41], making it difficult for consumers with BPD and their carers/families to receive treatment and support when needed [3, 4].

There are also concerns regarding the capacity of existing health services to meet the complex needs of consumers with BPD and their carers/families [34, 35, 41, 42]. This stems from the insufficient allocation of resources and funds to support BPD-related research, health service provision [41, 43, 44], education and training, and supervision for health practitioners working with consumers with BPD [45–55]. In addition, there are concerns regarding some practitioners’ stigmatizing beliefs, attitudes, and practices towards BPD [38, 39, 54]. Ungar et al.’s [56] study examined mental health practitioners’ beliefs and attitudes to treating consumers with BPD and found that more than 80% of staff agreed that consumers with BPD were more difficult to work with than consumers with other mental health disorders. Deans et al.’s [57] study found that 89% of psychiatric nurses (n = 47) agreed with the statement that consumers with BPD are ‘manipulative’. These findings are consistent with other studies exploring health practitioners’ perceptions of BPD [58, 59].

While there is vast literature on the perceptions and experiences of stigma among consumers with BPD [3, 9, 34, 60–72], their carers/families [1, 2, 4, 10, 11, 40, 73], and health practitioners [19, 38, 51–55, 57, 74–91], currently there is limited knowledge about the structural mechanisms contributing to BPD-related stigma within health systems, and their impact on the delivery of services and care to consumers with BPD and their carers/families. Exploring the body of literature addressing stigma in relation to BPD will allow us to identify the existing structural problems in healthcare systems and inform recommendations for addressing these significant public health concerns [29].

## Aim and research questions

The aim of this scoping review is to identify, map, and provide a broad overview of the international literature concerning structural stigma associated with BPD and its impact on healthcare for consumers with BPD, their carers/families, and health practitioners. This includes understanding how structures in health systems such as institutional policies, cultural norms, and organizational practices affect the availability and accessibility of quality health services and care for consumers with BPD and their carers/families. The primary research question addresses: How does structural stigma relevant to the diagnosis of BPD impact on the provision of health services and care for people with BPD, their carers/families, and health practitioners? Secondary research questions were also explored to gain a deeper understanding of the mechanisms, challenges, and barriers influencing BPD-related stigma in health systems. These were: (1) what are the perspectives and lived experiences of structural stigma among consumers with BPD, their carers/families, and health practitioners? (2) What are the specific drivers influencing the manifestation and perpetuation of BPD-related structural stigma in health systems, and the implications for research, policy, and practice? [92].

## Methods

The scoping review was registered within the Open Science Framework (registration ID: (https://osf.io/bhpg4). A scoping review methodology was chosen to achieve the aim of this review based on its broad application to mapping, exploring, and synthesizing extant international literature and identifying gaps in knowledge [93]. Scoping review approaches are useful for understanding the complexity of concepts relating to healthcare and informing evidence-based practice [94]. The review process followed JBI guidelines for scoping reviews [95] and Arksey and O’Malley’s [96] five-step framework for scoping reviews: (1) identifying the research question; (2) identifying relevant studies; (3) study selection; (4) charting data; and (5) collating, summarizing, and reporting the results.

### Identifying relevant studies

We undertook a comprehensive systematic search of electronic databases for peer-reviewed papers published from inception to February 28th 2022 using MEDLINE (Ovid), CINAHL (EBSCO Connect), PsycINFO (Ovid), Scopus (Elsevier), Cochrane Library (Wiley), and JBI Evidence-Based Database (Ovid). A search of grey literature using Google search engine was conducted to identify other relevant citations such as clinical practice guidelines for BPD. The references of included citations from both the peer-reviewed and grey literature searches were hand-searched to identify any additional relevant citations. Additional file 1 presents the PsycINFO search strategy and the grey literature key words. Search terms were developed as relevant to the three categories of key search terms: (a) BPD; (b) stigma; and (c) crisis care. Draft searches were executed in PsycINFO (Ovid) to test the search text word terms and subject heading combinations. Search terms were refined during iterative test searches resulting in a comprehensive search strategy to identify all existing peer-reviewed articles relating to BPD-related stigma associated with crisis presentations (i.e., a crisis relating to self-harm or suicidality) across various population groups (i.e., consumers with BPD, carers/families of people with BPD, and health practitioners) and healthcare settings (e.g., mental health and emergency services). Risk of selection bias was minimized by using multiple search methods. The eligibility criteria (Table 1), based on the Population-Concept-Context (PCC) framework [95], guided the study selection process during screening.

**Table 1:** Eligibility criteria

Population, concept, context	Criteria
Population	Health practitioners including, psychiatrists, psychologists, social workers, mental health nurses, general practitioners, primary care nurses, and other mental health workers who treat people with BPD in healthcare settings such as outpatients, inpatients, and community-based settings; people with BPD; carers/families of people with BPD
Concept	Structural stigma specific to BPD and crises (suicidality)
Context	International peer-reviewed studies investigating health practitioners’ attitudes and practice in treating people with BPD in healthcare settings; consumers with BPD or carers/families of people with BPD accessing healthcare
Inclusion criteria	Exclusion criteria
Articles were included if:	Articles were excluded if:
Evaluated health practitioners’ treating people with BPD in crisis in an outpatient, inpatient, and community-based setting; consumers with BPD or carers/families of people with BPD perspectives and experiences of healthcare	Evaluated health practitioners’ treating people with other mental illnesses; consumers with other mental illnesses; carers/families of people with other mental illness
Evaluated structural stigma as an outcome in healthcare settings	Not reporting outcomes specific to borderline personality disorder and structural stigma
Original research including peer-reviewed publications on quantitative, qualitative, mixed-methods, and review designs	Conducted in non-clinical settings such as educational institutions
Accessible for download free of charge	Studies of low quality
Written in English language only	

### Study selection

All citations identified from the comprehensive search were collated and uploaded into Endnote V.9. Citations were then uploaded into Covidence and de-duplicated by the lead author (PK). Citation screening and selection were undertaken using the Preferred Reporting Items for Systematic Reviews and Meta-Analyses (PRISMA) statement [97] (Additional file 2). Two independent reviewers (PK and AKF) screened the titles, abstracts, and full-text citations against the defined selection criteria to identify relevant studies. Full-text citations of selected studies were retrieved via Covidence and assessed against the inclusion criteria. Ineligible citations were omitted in accordance with the exclusion criteria (Table 1). Discrepancies in reviewer decisions regarding the inclusion of studies at both the title/abstract screening stage and the full-text stage were assessed and resolved by a third reviewer (SL) who had clinical expertise in mental health.

### Charting the data

The type of information to be extracted from the eligible citations was discussed and consensus reached following meetings held by the research team (PK, AKF, SL). Data identified for inclusion in this review were extracted into a charting table. The charting table of included studies used the following fields: author, year, country; quality rating; population data; aim/purpose; study design; methods; and main findings (Table 2). Data extraction was led by the first author (PK) and checked and revised by the second author (AKF).

**Table 2:** Data extraction of study characteristics on borderline personality disorder related structural stigma in healthcare systems

Author, Year, Country	MMAT v.18*/ JBI quality rating^a^	Population data	Aim/purpose	Study design	Methods/intervention	Main findings
Acres et al. 2019, AUS [10]	JBI, Level 1.b/ Level 4^b^	Carers (N = 1891). Emergency care settings. Sources of evidence (N = 10): research studies (n = 7), advocacy brief (n = 1), clinical practice guideline (n = 1), action plan (n = 1)	Explore, locate, and compile literature detailing the perspectives of family carers of people with BPD in Emergency Departments (ED) with a focus on nursing practices	Scoping review	Review of the literature	Carers perceived ED as the only option for emergency care in a crisis. Carers require information on how to manage a crisis with their loved one. Carers are often not consulted with by health professionals; and perceive that health professionals lack understanding of consumers distress and BPD—a key barrier to effective crisis care
Bodner et al. 2011, IL [76]	***	Health practitioners (N = 57): males (n = 35), females (n = 65) age, range (25–65 years old). Psychiatric hospital settings	Develop and use inventories that measure cognitive and emotional attitudes of health practitioners toward patients with BPD	Quantitative study	Surveys	Psychologists scored lower than psychiatrists and nurses on antagonistic judgments; nurses scored lower than psychiatrists and psychologists on empathy. Analyses conducted on the three emotional attitudes separately showed that suicidal tendencies of BPD patients explained negative emotions and difficulties in treating these patients
Bodner et al. 2015, IL [77]	****	Health practitioners (N = 710): age, range (40–47 years old). Years of service, range (11–21 years). Psychiatric hospitals (N = 4)	Improve Bodner et al. [76] sample; inspect if nurses’ tendency to express more negative attitudes toward BPD is evident in a larger sample	Quantitative study	Surveys	Nurses and psychiatrists reported higher number of patients with BPD, exhibited more negative attitudes, and less empathy toward these patients than other professions. Negative attitudes were positively correlated with caring for greater numbers of patients with BPD. Nurses expressed the greatest interest in studying short-term methods; psychiatrists expressed interest in improving professional skills for BPD
Borschmann et al. 2014, UK [7]	****	People with BPD (N = 41) males (n = 7, 17%), female (n = 34, 82%). Mean age (SD) 36, (11). Community services	Examine crisis care preferences of community-dwelling adults with BPD	Qualitative study	Thematic analysis of crisis plans	Participants gave clear statements in their crisis plans about the desire to recover from the crisis and improve their social functioning. Key themes included: the desire to be treated with dignity and respect; to receive emotional and practical support from clinicians; and preferences for treatment refusals during crises such as, psycho-tropic medication and involuntary treatment
Buteau et al. 2008 [1]	*****	Carers of people with BPD/ families (N = 12). Males (n = 2), and females (n = 10)	To learn from families what their experiences have been in four key areas: (1) knowledge about BPD, (2) BPD treatments, (3) coping with BPD, and (4) reasons for hope	Qualitative study	Semi-structured interviews	Families identified five key areas of concern: (1) difficulty accessing current evidence-based knowledge about BPD/ treatments, (2) a stigmatizing health care system, (3) prolonged hopelessness, (4) shrinking social networks, and, (5) financial burdens. To improve the quality of services for families affected by BPD, social workers must educate themselves on BPD, BPD treatment options, information, and resources
Carrotte et al. 2019, AUS [62]	*****	A total of 12 participants comprising, people with BPD (n = 9), carers (n = 3)	Identify treatment and support services accessed by people with BPD and their carers; perceived benefits and challenges associated with these services; and recommended changes to services	Qualitative study	Semi-structured interviews, focus groups	Themes revealed: identity and discovery; (mis)communication; complexities of care; finding what works; an uncertain future; and carer empowerment. Participants described community-based psychotherapy as critical for reducing symptoms of BPD and improving services. Macro- and micro-levels relating to costs, service access, and clinician-client factors were discussed
Clarke et al. 2015, UK [55]	****	Health practitioners (N = 44): years of service, range (1–10 years or more). Inpatient setting	To assess whether training in neurobiological underpinnings of BPD could improve knowledge and attitude change of staff	Within-subjects, quantitative survey design	Surveys relating to delivery of ‘The Science of BPD’ training	Attendance at the training session was associated with significant increases in theoretical knowledge, perspective taking and mental health locus of origin. There were no changes observed in empathic concern. A brief training session utilizing a neurobiological framework can be effective in facilitating knowledge and attitudinal change among health practitioners working with BPD
Commons Treloar et al. 2008, AUS [85]	****	Medical and health practitioners (N = 140). Males (n = 48), females (n = 92). Years of service, range (1–16 years). Emergency care, Mental health services settings	To assess the attitudes of clinicians towards patients diagnosed with BPD	Quantitative study	A purpose-designed survey	Significant differences were found among emergency medical and mental health staff in their attitudes to people with BPD. The strongest predictor of attitudes to self-harm were whether the practitioner worked in emergency medicine or mental health, years of experience, and training in BPD
Commons Treloar et al. 2009a, AUS [84]	*****	Medical and health practitioners (N = 140). Males (n = 48), females (n = 92). Emergency medicine, Mental health services settings (N = 3)	To explore health practitioners’ experiences and attitudes in working with patients with BPD	Qualitative study	Qualitative survey	Results revealed four themes: BPD patients generate an uncomfortable personal response in health practitioners, characteristics of BPD contribute to negative health practitioner/service responses, inadequacies of the health system in addressing BPD patient needs, and strategies needed to improve services for BPD. Findings suggest that interpersonal and system difficulties may have impacted the services for BPD
Commons Treloar et al. 2009b, AUS [53]	***	Registered health practitioners (N = 65). Males (n = 26, 40%), females (n = 39, 60%). Years of service (1 year or more. Psychiatric hospital settings	To examine two theoretical educational frameworks (cognitive-behavioural and psychoanalytic), compared with no education to assess subsequent differences in health practitioners’ attitudes to deliberate self-harm behaviours in BPD	A randomized comparative quasi-experimental study	Surveys/‘cognitive behavioural therapy program’, ‘psychotherapy program’ training	Compared with participants in the control group (N = 22), participants in the cognitive-behavioural program (N = 18) showed significant improvement in attitudes after attending the training, as did participants in the psychoanalytic program (N = 25). At six-month follow-up, the psychoanalytic group maintained significant changes in attitude. Results support the use of brief educational interventions in sustaining attitude change to working with this population
Day et al. 2018, AUS [86]	***	Mental health practitioners (N = 66). Males (n = 22, 33.3%), female (n = 44, 66.7%). Public health service settings	To investigate mental health practitioners’ attitudes to individuals with BPD where attitudes were compared over time	Longitudinal mixed methods design	Surveys, Semi-structured interviews	The 2000 sample (n = 33) endorsed more negative descriptions (e.g.,) ‘attention seeking’, ‘manipulative’), and the 2015 sample (n = 33) focused more on treatment approaches and skills (e.g.,) ‘management plan’, ‘empathy’). The 2015 sample endorsed more positive attitudes than the 2000 sample. This positive attitudinal shift may reflect a changing landscape of the mental health system and greater awareness and use of effective treatments
Deans et al. 2006, AUS [57]	****	Registered psychiatric nurses (N = 47). Males (n = 14, 30%), females (n = 34, 70%). Age, range (21–60 years old). 15 years or more (53%) of service. Psychiatric inpatient and community services	To describe psychiatric nurses’ attitudes to individuals with BPD	Quantitative study	Survey	Results show that a proportion of psychiatric nurses' experience negative reactions and attitudes to people with BPD, perceiving them as manipulative, and feeling angry towards them. One third of nurses reported they ‘strongly disagreed’ or ‘disagreed’ that they know how to care for people with BPD
Dickens et al. 2016, UK [52]	JBI, Level 1.b/ Level 4^b^	Mental health nurses (N = 1197). 9 studies across 6 Countries	To collate evidence on interventions devised to improve the responses of mental health nurses to people with BPD	Systematic Review	Review of the literature	Eight studies were included in this review, half of which were judged to be methodologically weak, and the remaining four studies judged to be of moderate quality. Only one study employed a control group. The largest effect sizes were found for changes related to cognitive attitudes including knowledge; smaller effect sizes were found in relation to changes in affective outcomes. Mental health nurses hold the poorest attitudes to people with BPD
Dickens et al. 2019, UK [51]	****	Mental health nurses (N = 28, training and pre-and post- surveys; N = 16, 4-month survey; N = 11, focus groups). Inpatient and community settings	To evaluate mental health nurses’ responses and experiences of an educational intervention for BPD	Mixed methods	Surveys, focus groups/‘positive about borderline’ training	Results revealed some sustained changes consistent with expected attitudinal gains in relation to the perceived treatment characteristics of this group, the perception of their suicidal tendencies and negative attitudes. Qualitative findings revealed hostility towards the underpinning biosocial model and positive appreciation for the involvement of an expert by experience
Dunne & Rogers, 2013, UK [2]	*****	Carers (N = 8). Community Personality Disorder Service	To explore carers’ experiences of the caring role, and mental health and community services	Qualitative study	Focus groups	The first carers’ focus group exploring the role of mental health services produced four super-ordinate themes. The second carers’ focus groups experiences in the community produced six super-ordinate themes. It seems carers of people with BPD are often overlooked by mental health services, and subsequently require more support to ensure their own well-being
Ekdahl et al. 2015, SE [73]	*****	Carers/ significant others (N = 19). Of the 19, 11 were involved in focus groups. Males (n = 5), females (n = 14). Age, range (43–75 years old). Psychiatric and health service settings	To describe significant others’ experiences of living close to a person with BPD and their experience of psychiatric care	Qualitative study	Qualitative survey, focus groups	Results revealed four categories: a life tiptoeing, powerlessness, guilt, and lifelong grief, feeling left out and abandoned, and lost trust. The first two categories describe the experience of living close to a person with BPD, and the last two categories describe encounters with psychiatric care
Fallon 2003, UK [65]	****	People with BPD (N = 7). Psychiatric services	To analyse the perspectives and lived experiences of participants with BPD contact with psychiatric services	Qualitative study	Unstructured interviews	Results found that people with BPD valued contact with psychiatric services despite negative staff attitudes and experiences. Relationships with others was vital in containing their distress despite trusting issues. Overcoming this was achieved by consistent long-term involvement with staff, containing relationships, encouraging participants to contribute to their care, and improving understanding of BPD
Hauck et al. 2013, USA [88]	****	Psychiatric nurses (N = 83) males (n = 8, 9.6%); females (n = 75, 90.3%). Age, range (21–65 years old). Psychiatric hospitals (N = 3)	To explore the attitudes of psychiatric nurses to patients with BPD experiencing self-harm	Descriptive, correlational design	Surveys	Psychiatric nurses had positive attitudes toward hospitalized BPD patients with deliberate self-harm. Psychiatric nurses with more years of nursing experience and self-reported need for further BPD education had more positive attitudes
Horn et al. 2007, UK [68]	*****	People with BPD (N = 5). Male (n = 1), female (n = 4). Age, range (23–44 years old). Mental health services	To explore user experiences and understandings of being given the diagnosis of BPD	Qualitative study	Semi-structured interviews	Analysis identified five themes: knowledge as power, uncertainty about what the diagnosis meant, diagnosis as rejection, diagnosis is about not fitting, hope and the possibility of change. Positive and negative aspects to these themes were apparent
James et al. 2007 IL [121]	***	Psychiatric nurses (N = 157) males (n = 21, 32%), females (n = 44, 68%) age, range (< 25- > 50) Years of service (< 2- > 15 years old). Various Psychiatric services	To describe the experiences and attitudes of nurses who deliver nursing care to people with BPD	Descriptive survey research design	Surveys	Results indicated that most nurses have regular contact with clients with BPD and nurses on inpatient units reported more frequent contact than nurses in the community. Eighty per cent of nurses viewed clients as more difficult to care for than other clients and believe that the care they receive is inadequate. Lack of services was the most important factor contributing to the inadequate care and the development of a specialist service as the most important to improve care
Keuroghlian et al. 2006, USA [50]	****	Medical and health practitioners (N = 297). Males (n = 25, 25.3%), females (n = 75, 74.7%) Mean years of service (SD) (17 years old (12). Medical centres, hospitals	To assess the effectiveness of Good Psychiatric Management workshops at improving clinicians’ attitudes to BPD; to assess if attitude changes relate to years of experience; and, compare the magnitude of change after GPM workshops to those from STEPPS workshop	Pre-post (repeated measures) design	Surveys/‘good psychiatric management’ training	Participants reported a decrease in the inclination to avoid, or dislike, patients with BPD, and belief that the prognosis is hopeless. were Participants also reported increased feelings of professional competence, belief that they can make a positive difference, and that effective psychotherapies do exist. Findings demonstrate Good Psychiatric Management’s potential for training health practitioners to meet the needs of people with BPD
Knaak et al. 2015, CA [49]	***	Health practitioners (N = 187) males (n = 28, 15%), female (n = 159, 85%. Mean age (39.1 years old). Mean years of service (22.2). Health services	To identify whether a generalist or specialist approach is the better strategy for anti-stigma programming for stigmatized disorders, and to examine the extent an intervention led to change in perceptions of people with BPD and mental illness	Pre-post design	Surveys/‘dialectical behaviour therapy’ training	Results suggest that the intervention was successful at improving healthcare provider attitudes and behavioural intentions towards persons with BPD. The results further suggest that anti-stigma interventions effective at combating stigma against a specific disorder may also have positive generalizable effects towards a broader set of mental illnesses
Koehne et al. 2012, AUS [19]	*****	Medical and health practitioners (N = 15). (Psychiatric hospitals (N = 3). Child and Adolescent Mental Health Services	Do mental health clinicians share diagnostic information about BPD with their adolescent clients, and if so, how? What are the factors that guide clinical practice in the decision to disclose or to withhold a diagnosis of emerging BPD to adolescents?	Qualitative study	Semi-structured interviews	Findings found that doctors, nurses, and allied health practitioners resisted a diagnosis of BPD in their work with adolescents. We delineate specific social and discursive strategies that health practitioners displayed including: team rules discouraged diagnostic disclosure, the lexical strategy of hedging when using the diagnosis, the prohibition and utility of informal ‘borderline talk’ among health practitioners reframed the diagnosis with young people
Lawn et al. 2015a, AUS [3]	***	People with BPD (N = 153). Age, range (18–65 years and older)	To explore the lived experiences of health service access from the perspective of Australians with BPD	Quantitative study	Survey	Responses from 153 consumers with BPD showed that they experience significant challenges and discrimination when accessing public and private health services. Seeking help from emergency departments during crises was challenging. Community support services were perceived as inadequate to meet patient needs
Lawn et al., 2015b, AUS [4]	***	Carers (N = 121). Males (n = 24, 25.5%), female (n = 78, 76.5%). Age, range (mostly 50-60 s). Various health and community services settings	To explore their experiences of being carers, attempts to seek help for the person diagnosed with BPD, and their own carer needs	Quantitative study	Surveys	Responses from 121 carers found carers experience significant challenges and discrimination when accessing health services. Comparison with consumers’ experiences showed that carers/families understand the discrimination faced by people BPD, largely because they also experience exclusion and discrimination. Community carer support services were perceived as inadequate. General Practitioners (GP) were an important source of support however, service providers need more education and training to support attitudinal change that addresses discrimination, recognizes carers’ needs, and provides support
Lohman 2017, USA [89]	*****	People with BPD (N = 500)/ BPD Resource Centre	To build on the BPD services knowledge base by characterizing the experiences of consumers, caregivers, and family members seeking BPD resources	Qualitative study	Retrospective data analysis of brief unstructured interviews (N = 500)/ Data from resource centre transcripts (N = 6253)	Results found that primary services and resources requested were: outpatient services (51%) and educational materials (13%). Care-seekers identified family services, crisis intervention, and mental health literacy as areas where available resources did not meet demand. Factors identified as potential barriers to accessing appropriate treatment for BPD included stigmatization and marginalization within mental health system and financial concerns
Ma et al. 2009, TW [109]	*****	Mental health nurses (N = 15). Females (n = 15). Age, range (20- > 40). Years of service (4–10 years). Various health and community service settings	To explore the contributing factors and effects of Taiwan’s mental health nurses’ decision-making patterns on care outcomes for patients with BPD	Qualitative study	Semi-structured interviews	The informants’ caring outcomes for BPD patients were involved with interactions across five themes: shifting from the honeymoon to chaos stage, nurses’ expectations for positive vs. negative outcomes, practicing routine vs. individualized care, adequate or inadequate support from healthcare teams and differences in care outcomes
Markham 2003, UK [104]	***	Mental health nurses (N = 71). Males (n = 18), females (n = 47). Mental health inpatient facilities	To evaluate the effects of the BPD label on staff attitudes and perceptions	Repeated measures factorial design	Surveys	Registered mental health nurses expressed less social rejection towards patients with schizophrenia and perceived them to be less dangerous than patients with BPD. Staff were least optimistic about patients with a BPD and were more negative about their experience of working with this group compared to the other patient groups
Markham et al. 2003, UK [122]	***	Mental health nurses (N = 48). Males (N = 12, 25%), females (N = 33, 69%). Mean age (SD), 38 (9.3). Mean years of service (SD), 12.7 (8.9). Mental health inpatient facilities	To investigate how the BPD label affects health practitioners’ perceptions and causal attributions about patients’ behaviour	Within-participants survey design	Survey	Patients with BPD attracted more negative responses from nurses than those with a label of schizophrenia. Causes of their behaviour were rated as more stable and they were thought to be more in control of their behaviour, then patients with other mental illnesses. Nurses reported less sympathy towards patients with BPD and rated their personal experiences as more negative than experienced with other patients
Masland et al. 2018, USA [48]	****	Mental health practitioners, researchers (N = 193). Mean age (SD), 48.84 (13.47). Mean years of service (SD), 18.12, (12.37). Various health services	To examine if the 1-day training can change health practitioners’ attitudes to BPD, which persist over time	Repeated measures design	Surveys/‘good psychiatric management’ training	Staff attitudes did not change immediately after training, but 6-months later had changed significantly. Findings indicated that brief training fosters improvements in health practitioners’ attitudes and beliefs about BPD
McGrath et al. 2012, IE [110]	*****	Registered psychiatric nurses (N = 17). Males (n = 5), females (n = 12). Mean years of service (n = 16). Community mental health service settings	To identify themes from an analysis of the nurses’ interactions with people with BPD, and to describe their level of empathy to this patient group	Qualitative study	Semi-structured interviews	Results found four themes: challenging and difficult, manipulative, destructive and threatening behaviour, preying on the vulnerable resulting in splitting staff and service users, and boundaries and structure. Low levels of empathy were evident in most participants’ responses to the staff-patient interaction response scale. Findings provide further insight on nurses’ empathy responses and views on caring for people with BPD
Millar 2012, SC [119]	****	Psychologists (N = 16). Females (n = 16). Years of service, range (1–32 years)	To explore psychologists’ experiences and perceptions of clients with BPD	Qualitative study	Focus groups	The following themes emerged from the analysis: negative perceptions of the client, undesirable feelings in the psychologist, positive perceptions of the client, desirable feelings in the psychologist, awareness of negativity, trying to make sense of the chaos, working in contrast to the system, and improving our role
Morris 2014, UK [111]	*****	People with BPD (N = 9). Males (n = 2), females (n = 7). Age, range (18–65 years old). Various voluntary sector organisations in the North-West of England	To explore people with BPD’s experience of mental health services to understand what aspects of services are helpful	Qualitative study	Semi-structured interviews	Three themes were generated including: the diagnostic process influences how service users feel about BPD, non-caring care, and it’s all about the relationship. Participants identified practical points which services could implement to improve the experiences of service users
National Health and Medical Research Council 2012, AUS [16]	Agree II Instrument level 6	Health practitioners	To provide current evidence for the effective treatment to improve the diagnosis and care of people with BPD in healthcare services in Australia	Clinical guidelines	Treatment and crisis management	Health professionals at all levels of the healthcare system and within each type of health service should recognize that BPD treatment is a legitimate use of healthcare services. Having BPD should never be used as a reason to refuse health care to a person. A tailored management plan, including crisis plan, should be developed for all people with BPD who are accessing health services
Nehls 1999, USA [112]	*****	People with BPD (N = 30). 30 Females (N = 30). Psychiatric, outpatient, and community services	To generate knowledge about the experience of living with the diagnosis of BPD	Qualitative study	Semi-structured interviews	Three themes were identified: living with a label, living with self-destructive behaviour perceived as manipulation, and living with limited access to care. The findings suggest that mental health care for persons with BPD could be improved by confronting prejudice, understanding self-harm, and safeguarding opportunities for dialogue
Nehls 2000, USA [113]	*****	Case managers (N = 17). Community mental health centre	To study the day-to-day experiences of case managers who care for persons with borderline personality disorder	Qualitative study	Semi-structured interviews	The analysis showed a pattern of monitoring self-involvement. The case managers monitored themselves in terms of expressing concern and setting boundaries. These practices highlight a central and unique component of being a case manager for persons with BPD, that is, the case manager's focus of attention is on self. By focusing on the self, case managers seek to retain control of the nature of the relationship
Ng 2016, AUS [123]	JBI, Level 1.b/ Level 4^b^	People with BPD (N = 1122), carers and health practitioners’ perspectives reflected in consumer studies	To review the literature on symptomatic and personal recovery from BPD	Systematic review	Review of the literature	There were 19 studies, representing 11 unique cohorts meeting the review criteria. There was a limited focus on personal recovery and the views of family and carers were absent from the literature. Stigma associated with the diagnostic label hindered trust formation and consumers ability to fully engage
O’Connell 2013, IE [120]	***	Community psychiatric nurses (N = 15). Years of service, range (3–15 years). Irish adult community mental health service	To explore the experience of psychiatric nurses who work in the community caring for clients with BPD	Qualitative study	Semi-structured interviews	The nurses’ understanding of BPD and their experiences of caring for individuals with the condition varied. Participants identified specific skills required when working with clients, but the absence of supervision for nurses was a particular difficulty, and training on BPD was lacking
Perseius et al. 2005, SE [8]	*****	People with BPD (N = 10) age, range (22–49 years old)	To investigate life situations, suffering, and perceptions of encounters with psychiatric care among patients with BPD	Qualitative study	Semi-structured interviews	Findings revealed three themes: life on the edge, the struggle for health and dignity, a balance act on a slack wire over a volcano, and the good and the bad act of psychiatric care in the drama of suffering. Theme formed movement back and forth, from despair and unendurable suffering to struggle for health and dignity and a life worth living
Pigot et al. 2019, AUS [47]	*****	Mental health practitioners (N = 21). Males (n=10), female (n = 11). Mean age, 39.5 (9.7). Public mental health services	To understand the facilitators and barriers to implementation of a stepped care approach to treating personality disorders	Qualitative study	Semi-structured interviews /‘stepped care approach’ training	Participants identified personal attitudes, knowledge, and skills as important for successful implementation. Existing positive attitudes and beliefs about treating people with a personality disorder contributed to the emergence of clinical champions. Training facilitated positive attitudes by justifying the psychological approach. Findings suggests specific organizational and individual factors may increase timely and efficient implementation of interventions for people with BPD
Proctor et al. 2020, AUS [106]	***	People with BPD (N = 577), comprising 153 consumers in 2011, and 424 consumers in 2017	To understand Australian consumer perspectives regarding BPD	Quantitative study	Surveys	Many people diagnosed with BPD experience difficulties when seeking help, stigma within health services, and barriers to treatment. Improved general awareness, communication, and understanding of BPD from consumers and health professionals were evident
Ring et al. 2019, AUS [34]	JBI, Level 1.b/ Level 4^b^	People with BPD (N = 12), Health practitioners (N = 18) across 30 studies in total	To compare and contrast what stigma looks like within mental health care contexts, from the perspective of patients and mental health professionals’ and how it is perpetuated at the interface of care	Literature review	Review of the literature	Thirty studies were found: 12 on patient’s perspectives and 18 on clinician’s perspectives. Six themes arose from the thematic synthesis: stigma related to diagnosis and disclosure, perceived un-treatability, stigma as a response to feeling powerless, stigma due to preconceptions of patients, low BPD health literacy, and overcoming stigma through enhanced empathy. A conceptual framework for explaining the perpetuation of stigma and BPD is proposed
Rogers 2012, UK [69]	*****	People with BPD (N = 7). Male (n = 1), female (n = 5) age, range (22–66 years old)	To explore the experience of service users being treated with medication for the BPD diagnosis	Qualitative study	Semi-structured interviews	The main themes to emerged were: staff knowledge and attitudes, lack of resources and the recovery pathway for BPD. Service users felt that receiving the BPD diagnosis had a negative impact on the care they received, with staff either refusing treatment or focusing on medication as a treatment option. The introduction of specialist services for this group appears to improve service user satisfaction with treatment and adherence to the National Institute for Clinical Excellence guidelines
Shaikh et al. 2017, USA [33]	JBI, Level 1.b/ Level 4	Health practitioners (N = 5136). 56 studies. Emergency care	To review the advice to physicians and health-care providers who face challenging BPD patients in the ED	Systematic review	Review of the literature	Results found that crisis intervention should be the first objective of health practitioners when dealing with these patients in emergency departments. Risk management processes and developing a positive attitude and empathy towards these patients will help them in normalizing in an emergency setting after which treatment course can be decided
Sitsti 2016, USA [108]	****	Psychiatrists (N = 134). Male (n = 88, 65.7%), females, (n = 46, 34.3%). Years of service, range (0- > 20). Psychiatric services	To examine whether Psychiatrists had ever withheld/not documented patients’ BPD diagnosis	Quantitative study	Survey	Fifty-seven percent of participants indicated that they failed to disclose BPD to their patients, and 37 percent said they had not documented the diagnosis. For those respondents with a history of not disclosing or documenting BPD, most agreed that either stigma or uncertainty of diagnosis played a role in decisions
Stapleton et al. 2019, UK [71]	JBI, Level 1.b/ Level 4	People with BPD (N = 90) across all 8 studies. Age, range between 21 and 61 years. Acute Psychiatric inpatient wards	To conduct a meta-synthesis of qualitative research exploring the experiences of people with BPD on acute psychiatric inpatient wards	Meta-synthesis	Review of the literature	Eight primary studies met the inclusion criteria. Four themes included: contact with staff and fellow inpatients, staff attitudes and knowledge, admission as a refuge, and the admission and discharge journey. Opportunities to be listened to and to talk to staff and fellow inpatients, time-out from daily life and feelings of safety and control were perceived as positive elements of inpatient care. Negative experiences were attributed to a lack of contact with staff, negative staff attitudes, staff’s lack of knowledge on BPD, coercive involuntary admission, and poor discharge planning
Stroud et al. 2013, UK [39]	*****	Registered Community Mental Health Nurses (N = 4). Male (n = 1, female (n = 3). Age range (30–59 years old). Community Mental Health team	To gain a fuller understanding of how community psychiatric nurses make sense of the diagnosis of BPD and how their constructs of BPD impact their approach to this client group	Qualitative study	Semi-structured interviews	Results suggested that participants ascribe meaning to the client’s presentation ‘in the moment’. When they had a framework to explain behaviour, participants were more likely to express positive attitudes. As participants were deriving meaning ‘in the moment’, there could be fluidity with regards to participants’ attitudes, ranging from ‘dread’ to a ‘desire to help’, and leading participants to shift between ‘connected’ and ‘disconnected’ interactions
Sulzer 2015, USA [114]	*****	Mental health practitioners (N = 22). Inpatient and out-patient settings	To evaluate how health practitioners describe patients with BPD, how the diagnosis affects the treatment provided, and the implications for patients	Qualitative study	Semi-structured interviews	Findings suggest patients with BPD are routinely labelled difficult, and subsequently routed out of care through a variety of direct and indirect means. This process creates a functional form of demedicalization where the actual diagnosis of BPD remains de jure medicalized, but the treatment component of medicalization is harder to secure for patients
Sulzer 2016a, USA [115]	*****	Mental health practitioners (N = 22). BPD activists	To understand how health practitioners communicate the diagnosis of BPD with patients, and to compare and evaluate these practices with patient communication preferences	Qualitative study	Semi-structured interviews	Most participants sampled did not actively share the BPD diagnosis with their patients, even when they felt it was the most appropriate diagnosis. Most patients wanted to be told that they had the disorder, as well as have their providers discuss the stigma they would face. Patients who later discovered that their diagnosis had been withheld consistently left treatment
Sulzer 2016b, USA [118]	****	Mental health practitioners (N = 39). Men (n = 15), female (n = 24). Various public and private health services	To examine how clinicians navigate providing treatment to BPD in the context of the DSM 5, deinstitutionalization, and the biomedical model	Qualitative study	Semi-structured interviews	Health practitioners faced pressures to focus on biomedical treatments. Treatments which emphasized pharmaceuticals and short courses of care were ill-suited compared to long-term therapeutic interventions. This contradiction is the ‘biomedical mismatch’; Gidden's concept of structuration is used to understand how health practitioners navigate care. Social factors such as, stigma and trauma, are insufficiently represented in the biomedical model of care for BPD
National Institute for Health and Care Excellence, 2009 UK [107]	Agree II Instrument level 5	Targeting Health practitioners	To advise on the treatment and management of BPD	Clinical guidelines	Treatment and crisis management	Findings provide evidence-based guidance on interventions for health practitioners supporting people with BPD and families/carers. People with BPD should not be excluded from accessing health services because of their diagnosis or suicidality. Health practitioners should build a trusting relationship, work in an open, engaging, and non-judgmental manner, and be consistent and reliable when working with people with BPD and carers
Vandyk et al. 2019, CA [117]	*****	People with BPD (N = 6). Emergency care settings	To explore the experiences of persons who frequently present to the ED for mental health-related reasons	Qualitative study	Semi-structured interviews	Two broad themes included: the cyclic nature of ED use, coping skills and strategies. Unstable community management that leads to crisis presentation to the ED often perpetuated access by participants. Participants identified a desire for human interaction, feelings of loneliness, lack of community resources, safety concerns following suicidality as the main drivers for visiting ED. Participants identified strategies to protect themselves against unnecessary ED use and improve health
Veysey 2014, NZ [72]	*****	People with a BPD (N = 8). Male (n = 2), female (n = 7). Age, range (25–65)	To explore people with BPD encounters of discriminatory experiences from healthcare professionals	Qualitative study	Semi-structured interviews	Themes found that discriminatory experiences contributed to participants’ negative self-image and negative messages about the BPD label. A history of self-harm appeared to be related to an increased number of discriminatory experiences. Connecting with the person and ‘seeing more’ beyond an individual’s diagnosis and/or behaviour epitomized helpful experiences
Warrender 2015, UK [45]	*****	Nurses (N = 9). Acute mental health wards, hospital setting (N = 1). Health services	To capture staff perceptions of the impact of health. Mentalization-based therapy skills training on their practice when working with people BPD in acute mental health	Qualitative study	Focus groups/‘mentalization-based therapy skills training	Mentalization-based Therapy Skills training promoted empathy and humane responses to self-harm, impacted on participants ability to tolerate risk and changed some perceptions of BPD. Staff felt empowered and more confident working with people with BPD. The positive implication for practice was the ease in which the approach was adopted and participants perception of Mentalization-based Therapy skills as an empowering skill set which also contributed to attitudinal change
Warrender et al. 2020, UK [35]	JBI, Level 1.b/ Level 4	Health practitioners 46 studies. A total of N = 3714 participants comprising: people with BPD (n = 2345), carers (n = 184), Health practitioners (n = 1185). Various healthcare settings	To explore the experiences of stakeholders involved in the crisis care of people diagnosed with BPD	Integrative review	Systematic review of the literature	Four themes: crisis as a recurrent multidimensional cycle, variations and dynamics impacting on crisis intervention, impact of interpersonal dynamics and communication on crisis, and balancing decision making and responsibility in managing crisis
Wlodarczyk et al. 2018 AUS [42]	*****	A total of 22 participants comprising GP (N = 12); research team (n = 5); People with BPD (n = 2); Carers, 3. GPs: males (n = 6), females (n = 6). GP Partners Australia	To explore the nature and difficulties for GP, examine the reasons that caring for people with BPD in primary care is difficult and not well managed, and explore what strategies and actions might assist with improving the care of their patients with BPD	Qualitative study	Focus groups	Key themes identified were: challenges regarding the BPD diagnosis, clinical complexity, the GP–patient relationship, and navigating systems for support. Health service pathways are dependent on the quality of care provided and GP capacity to identify and understand BPD. GP need support to develop the skills necessary to provide effective care for BPD patients. Structural barriers obstructing attempts to address patients with BPD were discussed
Woollaston et al. 2008, UK [116]	*****	Nurses (N = 6). Males (n = 4), females (n = 2). Age, range (20–40 years old). Years of service, range (2–17 years). Various hospital and community health services (N = 6)	To explore nurses’ relationships with BPD patients from their own perspective	Qualitative study	Semi-structured interviews	Results identified the following themes: destructive whirlwind’, idealized and demonized, and manipulation and threatening. The study concludes that nurses experience BPD patients negatively. This can be attributed to the unpleasant interactions they have with them and feeling that they lack the necessary skills to work with this group. Nurses report that they want to improve their relationships with BPD patients

*BPD* Borderline Personality Disorder, *ED* Emergency department

^a^MMAT v.18 quality rating: low = 1* to 2** stars; moderate = 3*** stars; moderately high = 4**** stars; high = 5***** stars[97]

^b^JBI Quality rating for level of evidence for effectiveness is level 1.b systematic review of RCTs and other study designs; and the level of meaningfulness is 4—systematic reviews of expert opinion[120]

^c^Agree II Instrument quality rating scale [99]: 1 = lowest possible quality, through to, 7 = highest possible quality

### Quality appraisal

Quality appraisal of all citations was undertaken to reduce the risk of bias. The MMAT v.18 checklist [98] was used to determine methodological quality of the quantitative, qualitative and mixed methods studies for inclusion in this review. The JBI Checklist for Systematic Reviews and Research Syntheses tool [99] was used to appraise methodological rigor of the reviews; the AGREE II: advancing guideline development, reporting, and evaluation in health care tool [100] was used to appraise the Clinical Practice Guidelines for the management of BPD (referred to as Guidelines) [16]. Meetings were held by the research team (PK, AKF, SL) to discuss the application of items within the quality appraisal tools and processes for assessing the methodological quality of the included citations. This included establishing an agreed cut-off criteria to exclude low quality studies in accordance with the eligibility criteria. Initially, one reviewer (PK) conducted the quality appraisals of the citations. Two reviewers (PK and AKF) then met to review the quality appraisals of the studies and highlight any concerns; where issues were identified, resolution was achieved through discussion. Although a third reviewer (SL) was available to resolve any discrepancies, no further resolution was required.

### Collating and summarizing findings

Data were collated, analysed, and synthesized using Braun and Clarke’s [101] Thematic Analysis. Results of the review were synthesized into a narrative summary of the study aims, research questions, and eligibility criteria (PCC). Data analyses involved: (1) quantitative data being summarized using descriptive statistics and frequencies [102]; and (2) Thematic Analysis of qualitative data to organize, categorize, and interpret key themes and patterns emerging from the data [101]. Trustworthiness and rigor of data abstraction and synthesis were established using a data analysis table that captured the categories, codes, and key findings/themes on the impact of structural stigma on healthcare for consumers with BPD, their carers/families, and health practitioners. Triangulating the perspectives and lived experiences of the relevant populations (i.e., consumers with BPD, their carers/families, and health practitioners) has been identified as an effective approach to establishing a comprehensive understanding of the complex nature of health systems [103].

## Results

### Data characteristics

**Fig. 1:**
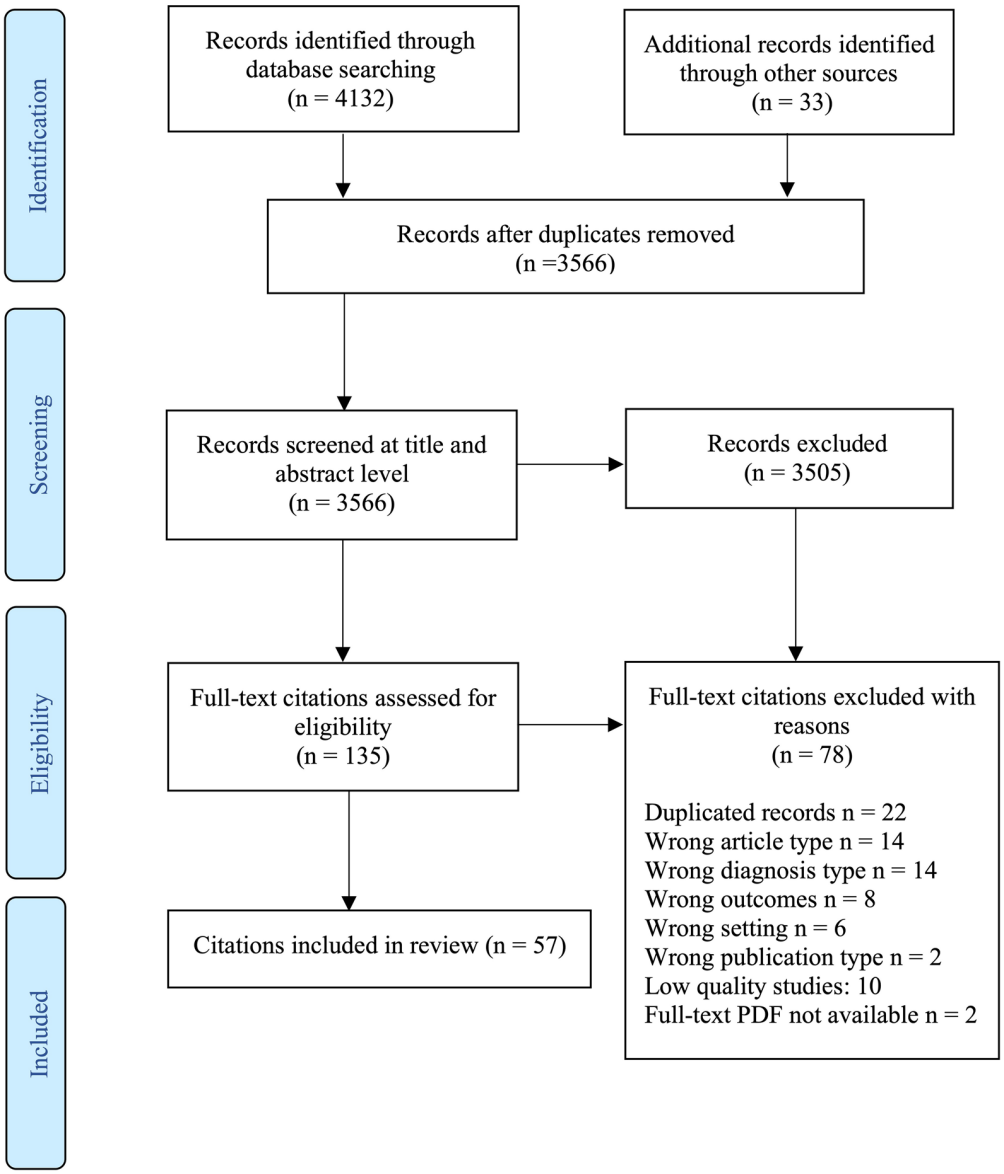
PRISMA flowchart of the selection of citations for the scoping review

The initial database searches yielded 4132 publications. An additional 33 (n = 33) records were identified via other sources. Following the removal of duplicates, citation titles and abstracts were screened (n = 3566), and full-text records (n = 135) were retrieved and assessed for eligibility. Of these records, 78 (n = 78) were excluded when assessed against the inclusion criteria and the quality appraisal criteria. In total, 57 (n = 57) citations that aligned with the inclusion criteria and study aims were incorporated into this review. Search results including reasons citations were excluded are presented in a PRISMA Flow Diagram (Fig. 1). Most of the citations comprised peer-reviewed published studies (n = 55), and two (n = 2) non-published reports. The majority of the citations examined health practitioners' stigmatizing attitudes and practice specific to BPD (n = 36, 63%). Some citations focused on BPD-related educational interventions designed to modify health practitioners' attitudes and practice in treating BPD (n = 9, 5%) [45, 47–53, 55]. Table 2 presents data characteristics of included citations. Table 3 summarizes study characteristics of included studies.

**Table 3:** Summary of key study characteristics

	N	%
Participants	17,406	100
Study methodologies		
Quantitative studies	18	32.0
Qualitative studies	28	49.0
Mixed methods study	2	3.5
Reviews/meta-synthesis	7	12.0
Clinical practice guidelines	2	3.5
Countries		
Australia	16	28.0
Israel	3	5.0
United Kingdom	17	30.0
Sweden	2	3.3
United States of America	11	19.0
Canada	2	3.3
New Zealand	1	2.0
Ireland	2	3.3
Scotland	1	2.0
Taiwan	1	2.0
Unknown	1	2.0
Population groups^a^		
Health practitioners	37	65.0
Consumers with BPD	15	25.0
Carers/families	6	10.0
Healthcare settings		
Mental health services	19	31.0
Emergency departments	13	21.0
General hospital and health services	14	24.0
Community-based services	14	24.0
Health professions		
Medicine/psychiatry	18	30.0
Nursing	26	42.0
Allied health	17	28.0

^a^Some citations included more than one population group, healthcare setting, and health profession

## Methodological quality

Critical appraisal of all included citations found that the quality ratings of the quantitative studies were moderate (n = 9), [3, 4, 49, 53, 76, 104–107], and moderately high (n = 8) [48, 50, 55, 57, 77, 85, 88, 108]. The quality of most qualitative studies was rated as high (n = 24) [1, 2, 8, 19, 39, 42, 45, 47, 62, 68, 69, 72, 73, 84, 89, 109–117], or moderately high in quality (n = 4) [7, 65, 118, 119]. One (n = 1) qualitative study was deemed moderate in quality [120]. The quality of the mixed methods studies (n = 2) was determined as moderate [86] and moderately high [51].

Reviews were of moderate (n = 6) [10, 33, 35, 52, 71, 123], or high quality (n = 1) [34], and reports were moderately high in quality (n = 2) [16, 107] (Table 2).

## Key findings

Synthesis of the review findings identified several extant macro- and micro-level structural mechanisms, challenges, and barriers associated with BPD-related stigma in health systems. Structural problems that contribute to BPD-related stigma were evident across multiple levels of the health sector including system/service-, practitioner-, and consumer-levels. These results highlight the complex and contentious nature of BPD and healthcare across the following broad themes (and sub-themes) comprising: structural stigma and the BPD diagnosis (subthemes—legitimacy of the BPD diagnosis, reluctance to disclose a BPD diagnosis, discourse of untreatability); BPD-related stigma surrounding health and crisis care services (subthemes—BPD and healthcare, practitioner-consumer interactions). Each of these themes and subthemes are discussed below.

### Structural stigma and the BPD diagnosis

This theme is centred around the dominant stigma discourse and misconceptions within health systems regarding the BPD diagnosis, its disclosure to the consumer, treatment options, and prognosis for recovery from the perspectives of health practitioners [16, 19, 33, 34, 108, 114, 115, 118, 119, 123], consumers with BPD [3, 34, 62, 68, 111, 112, 123], and carers/families [4, 10, 62, 123]. The main structural challenges and barriers associated with the diagnosis of BPD in health systems include: uncertainty whether BPD is a legitimate mental illness [19, 84, 114–116, 118]; concerns regarding the disclosure of a BPD diagnosis [65, 108, 115]; and perceptions that BPD is an untreatable condition [114, 116, 118]. Consequently, consumers with BPD are often denied evidence-based treatment [3, 4, 114, 115, 118] and routed out of the health system through a process called de-medicalization—making it difficult for these consumers to access health services and support [114].

#### Legitimacy of the BPD diagnosis

The BPD diagnosis and its legitimacy as a serious mental illness is actively contested in some health systems [19, 35, 114], which can create barriers to consumers with BPD accessing health services [16, 107]. Sulzer et al.’s [114] qualitative study found that some health practitioners viewed consumers with BPD from a moral stance, rather than the consumer being genuinely *unwell*; and consequently denied these consumers treatment. For instance, participants described consumers with BPD as morally deviant and viewed self-harming behaviour as attention-seeking, rather than perceiving it as a symptom of their underlying mental illness and associated distress. Nehls et al.’s [112] qualitative study suggest that some health practitioners held misconceptions regarding the disorder such as associating it with a *flaw in character*, rather than it being a legitimate *illness*. Health practitioners also believed consumers with BPD were responsible for their presentation and more in control of their actions than consumers with other mental health conditions [112, 114]. These misconceptions regarding the validity and reliability of the BPD diagnosis historically stem partly from the separation of BPD (and all personality disorders) into Axis II of the Diagnostic and Statistical Manual of Mental Disorders (DSM) [12]. This distinguished BPD from other mental health disorders such as schizophrenia which we understand has a clear biological aetiology and response to psychoactive medication [114]. Psychiatrists working with adolescents in Child and Mental Health Services also expressed concerns regarding the legitimacy of the BPD diagnosis for adolescents given the DSM criteria are adult-specific and do not account for the developmental stages of adolescents [19].

Consumers with BPD [3, 7, 65, 69, 71, 72, 112, 117] and their carers/families [1, 2, 4, 10, 42, 62, 73, 123] have consistently reported experiencing discrimination and stigma from health services in response to the BPD diagnosis. Lawn et al.’s [3, 4] quantitative studies found that consumers with BPD reported experiencing high levels of anxiety associated with ‘discrimination due to their BPD diagnosis’ (58%, n = 67) and ‘not being taken seriously’ (71%, n = 82) by health practitioners. Carers/families of people with BPD also reported that they were not taken seriously by practitioners (60.5%, n = 46/77). Carers/families also perceived the discrimination towards the BPD diagnosis (53%, n = 36), and not being taken seriously (44%, n = 30) as major barriers to accessing health services and support. These discriminatory experiences are contrary to best practice guidelines for the treatment and management of BPD [16, 107] which describes the disorder as a valid mental illness with effective treatments available and a legitimate use of healthcare resources. These guidelines also advise against discrimination or withholding treatment based on the presence of a BPD diagnosis.

#### Reluctance to disclose a BPD diagnosis

Studies examining BPD-related stigma in health systems have highlighted some health practitioners reluctance to disclose a BPD diagnosis to consumers [33, 34, 108, 114, 118, 123]. Sisti and colleague [108] undertook a quantitative survey and found more than half of psychiatrists (57%, n = 77) participating in the study chose not to disclose a BPD diagnosis to their patients; over a third of psychiatrists (37%, n = 49) did not document the diagnosis in their patient’s medical charts. Respondents in this study reported stigma (43%) and uncertainty regarding the diagnosis (60%) as the reasons for withholding a BPD diagnosis from patients. Respondents (n = 12) in Lawn et al.’s [3] survey suggested that family doctors (General Practitioners) did not appear to take notes on BPD or recognize the disorder. Koehne et al.’s [19] qualitative study explored health practitioners diagnostic and disclosure practices for adolescent patients and found that practitioners decisions regarding diagnostic disclosure were often influenced by cultural norms embedded within their professional teams. Findings further indicated that some health practitioners may use discursive strategies to avoid disclosing the diagnosis to their patients such as hedging (i.e., vague terms used to distance from the discussion at hand) and reframing the condition in terms of emerging traits, rather than naming the diagnosis.

Similarly, Sulzer et al. [115] found most health practitioners (81%, n = 32) diagnosed patients with an alternative disorder such as post-traumatic stress disorder or depression. Practitioners' reported reasons for providing patients with an alternative diagnosis included: fear of patient rejecting the diagnosis; protecting patient from stigma, shame, and blame associated with the disorder; and, enhancing patients' likelihood of receiving appropriate treatment for their mental health condition. These findings are consistent with consumer responses reported in this study, which indicated that they had not been informed about the BPD diagnosis by their health practitioner at the time of the diagnosis. Only a few health practitioners (9%) reported fully disclosing a BPD diagnosis to their patients. The reasons these participants gave for disclosing the diagnosis was to ensure that they were complying with their professional duties regarding informed consent for treatment, and to enable consumers to access appropriate treatment for their specific needs.

Contrary to health practitioners beliefs, some consumers with BPD in Sulzer et al.’s [115] study (n = 29) reported that they wanted to be informed of their diagnosis and discuss the disorder and its associated stigma with their health practitioner. Most consumers stated that they experienced relief when they received the diagnosis, and that they found the diagnostic process therapeutic. Few consumers (n = 3) reacted negatively to receiving a BPD diagnosis. These findings are consistent with other studies reporting that consumers with BPD appreciate being informed about the diagnosis by their health practitioner [34, 62]. In addition, Morris et al.’s [111] qualitative study suggested that the way in which people are diagnosed and informed about the diagnosis impacts how they feel about BPD. Consumers who were informed of their BPD diagnosis by a health practitioner that was optimistic about effective treatment and recovery prospects were more likely to feel positive about BPD than consumers who had a negative experience learning about their diagnosis. Further, Sulzer et al. [115] observed that some consumers with BPD whose health practitioner did not openly discuss their diagnosis with them subsequently disengaged with treatment.

Consumers with BPD also reported that receiving BPD-related information and education from their health practitioner was helpful [111, 112] as it assisted them to understand their symptoms and behaviours [65], and to see their condition from a disease perspective rather than as a personality flaw [34]. Other studies highlighted the importance of receiving adequate information about BPD from health practitioners; consumers who did not receive sufficient education showed limited knowledge and understanding of BPD [3, 68]. This experience may be relatively common given the findings of Lawn et al. [3] that 37.8% (n = 45/119) of consumers with BPD reported that they had not received any information from practitioners about what the BPD diagnosis means, and 19.3% (n = 23) of respondents stated that the diagnosis had been explained but they had not understood the information provided. These practices present major structural challenges and barriers to consumers with BPD as they prevent them from having adequate knowledge with which to understand their symptoms, as well as knowledge regarding treatment options to support them with their specific needs [16, 107].

#### Discourse of untreatability

The dominant biomedical approach to healthcare has been identified as an important structural mechanism driving the challenges and barriers to responsive services and care for BPD. Debates in the literature regarding the effectiveness of biomedical approaches for treating BPD, which rely upon conventional treatments such as medication and short-term intervention rather than longer-term therapy and support, are viewed by many health practitioners as ill-suited [118, 119]. Social factors contributing to stigma and trauma are not considered in the biomedical approach, and have consequently created the unintentional downstream effects of short-term crisis interventions, repetitive crisis presentations, and readmissions to hospital [118]. The inadequacy of the biomedical model to effectively respond to the complex needs of consumers with BPD is evident from their high rates of health service utilization, predominantly primary, emergency, and mental health services [118, 123]. However, health practitioners working with this population experience considerable pressure to align their practice to the dominant biomedical approach [118], despite the challenges associated with treatment, perceptions of poor prognosis, and recovery pathways for BPD [16, 34, 35, 39, 50–53, 57, 76, 77, 84–86, 104, 105, 107, 109, 110, 113, 116, 118, 119, 122].

Sulzer et al.’s [118] qualitative study suggests that some health practitioners avoid working with consumers with BPD based on the misconception that BPD is not treatable as it is not responsive to psychotropic medication. Another qualitative study [84] found that some health practitioners were less likely to provide an objective assessment of consumers' needs when BPD was present, and refused to treat consumers with BPD. Alarmingly, a few health practitioners (n = 2) [110] revealed that they avoided providing any (or minimal) level of care to consumers with BPD. These findings are consistent with reports of health practitioners witnessing their colleagues refusing to treat consumers with BPD [84]. Consistent with this, several studies (n = 9) reported consumers’ with BPD [3, 7, 62, 65, 68, 89, 111, 112, 118] and carers/families of people with BPD [4] being denied treatment by health practitioners when attempting to access health services and support. Consumers with BPD described their experiences of being denied treatment by health practitioners as distressing [16, 106], and that health practitioners’ preconceived ideas and attitudes to BPD left them feeling "labelled and judged" rather than, "diagnosed and treated" for their mental health condition ([112] p.288). Consumers with BPD also stated that although they believed receiving a diagnosis of BPD was useful in guiding treatment at times, the BPD diagnosis affected their treatment and recovery prospects [112]. These findings suggest that the myths concerning the untreatable nature of BPD persists to impact practice (i.e., denying treatment) despite evidence of effective treatments for BPD such as Dialectical Behavioural Therapy, Cognitive Behavioural Therapy, and mentalization based approaches [16, 107, 114].

### BPD-related stigma surrounding health and crisis care services

#### BPD and healthcare

Consumers with BPD and their carers/families frequently access health services in crisis [3, 4, 10, 65] and consequently, experience widespread discrimination, prejudice, and stigma in health systems [1–4, 7, 8, 10, 16, 19, 33–35, 39, 42, 45, 47–53, 55, 57, 62, 65, 68, 69, 71–73, 76, 77, 84–86, 88, 89, 104, 106–123]. A recent review described BPD-related crises as a recurrent multidimensional cycle of suicidality, help-seeking, and health service utilization, linked to the experience of distress among consumers with BPD, their carers/families, and health practitioners [35]. The experience of crisis for people with BPD has been described by consumers as a sudden onset of overwhelming emotions in response to negative internal and/or external stimuli such as interpersonal conflict associated with feelings of depression or anxiety. Experiences of intense emotional distress may lead to maladaptive coping strategies (e.g., feeling unsafe, self-harming behaviours, suicidality) and precipitate the involvement of emergency services.

A large-scale quantitative survey [3] undertaken in Australia supported previous evidence [12] that people diagnosed with BPD frequently experience high rates of suicidality. Survey results of the 99 consumer respondents who answered questions relating to suicidality found that 97% (n = 96/99) reported that they have had thoughts about self-harming, 98.9% (n = 94/95) of respondents reported that they had engaged in self-harming behaviours, 100% (n = 99) of participants reported having had thoughts of ending their lives, and 85.6% (n = 83/97) of respondents had made a previous serious attempt at ending their life. Carers/families of people with BPD also experience distress [35], and feelings of intense stress and worry in relation to the wellbeing and safety of the person that they care for when that person is experiencing crises [4, 35, 73]. Carers/families further disclosed that they experience feelings of hopelessness and social isolation when attempting to gain support from family [1] and health practitioners [1, 2, 4, 10].

Health practitioners similarly reported experiencing distress when working with consumers with BPD in crisis [35, 45] where the threat of suicide is considered to be the most distressing presentation [110]. Practitioners disclosed that they find treating consumers with BPD experiencing suicidality confronting [85]. Suicidality has historically been judged harshly by some health practitioners [89] viewing consumers with BPD who engaged in self-harming behaviour as *acting out* [8] to gain attention [8, 110] or control others [57]. In contrast, consumers with BPD argued that they are misunderstood by health practitioners as their self-harming behaviour is not intended to gain attention or control people; rather, it is a method for releasing or distracting from intense emotional pain [3, 8]. When recounting their impulsive self-harming behaviour, consumers with BPD reported feeling remorse following self-harm or a suicide attempt and then seeking help from health services [58] hoping to recover from the crisis and stabilize their mental health condition [7]. Although health practitioners understood that consumers with BPD engaged in self-harming behaviour in response to emotional distress, they considered these behaviours by consumers with BPD to be a habitual response [85]. The findings in relation to health practitioners responses to BPD and suicidality may contribute to the pervasive stigmatizing attitudes and practices displayed by some health practitioners [1–4, 7, 8, 10, 16, 19, 33–35, 39, 42, 45, 47–53, 55, 57, 62, 65, 68, 69, 71–73, 76, 77, 84–86, 88, 89, 104, 106–123].

Health practitioners feelings of frustration, inadequacy [84], and uncertainty regarding the treatment and management of consumers with BPD when they present to health services in crisis [45, 76, 77, 110] might be linked to a lack of knowledge, confidence, and skills to deliver high quality care that meet the complex needs of this population [47–53, 55, 57, 76, 78, 88]. Deans et al.’s [57] quantitative study revealed that one third of nurses who participated in the study (34%) reported that they did not know how to care for consumers with BPD. Respondents in Lawn et al.’s [3] study stated that their family doctor did not appear confident in treating BPD. These findings highlight the need for BPD-specific education, training, and supervision to assist health practitioners to better understand BPD and effectively treat consumers with this disorder [84, 89]. A recent review [124] found promising results regarding the capacity of BPD-specific educational interventions positively modifying health practitioners’ attitudes and practice to BPD. Health practitioners also reported that they wanted to receive education and training in BPD and crisis interventions to help them deliver better services and care to consumers with BPD [124]; practitioners perceived crisis as a consistent feature among consumers with BPD [69, 84, 113], often necessitating crisis intervention and referrals to intensive home treatment teams [35] or other community-based services and supports [3, 4].

Stigma in relation to BPD and suicidality has been identified in the literature as a pivotal structural mechanism underscoring the inadequacy of health and crisis care services and supports for BPD [3, 4, 62]. Studies [84, 121] found that health practitioners themselves reported that consumers with BPD and their carers/families receive inadequate care from health services. Associated challenges and barriers include the significant gaps in the availability, accessibility, and affordability of services and supports for consumers with BPD and their carers/families [3, 4, 8, 33, 35, 42, 65, 72, 73, 84, 85, 106, 109, 111, 112, 117, 120, 123]. Lawn et al.’s [3] studies found that 50% of consumers with BPD reported that they were unable to access support services when they needed them; 63% (n = 48) of carers/families [4] reported that they could not access support services, with 51.4% (n = 37) reporting that their family doctor had not supported them in their caring role.

Primary health care providers reported difficulties navigating health services and referral pathways for clients with BPD and carers/families given the limited services and supports available [42]. Hospital and community-based services and supports for people with BPD and carers/families in the public system is limited, with long waiting times for psychotherapy and other BPD-related programs [3, 4, 42]. Insufficient staff-to-patient ratios and time constraints to meet workload demands are also major barriers to delivering responsive services and care for BPD in emergency and mental health services [84]. In addition, financial barriers relating to the expense of accessing private specialist services often place significant economic demands on consumers with BPD and their carers/families [3, 4, 73]. These gaps in service provision for consumers with BPD place pressure on emergency departments as consumers with BPD and their carers/families perceive there to be few other service options [10, 117]. Consumers with BPD report viewing hospital admissions as a refuge and means by which to keep themselves safe due to the limited availability of community services and supports when experiencing suicidal thoughts and behaviours [71].

Despite ongoing attempts by consumers with BPD and their carers/families to seek help from hospitals for suicidality, there are conflicting views among health practitioners as to whether, or not, hospital admissions are effective for consumers with BPD at risk of suicide [16, 35, 57, 77, 84, 86, 107, 110]. While existing guidelines recommend that consumers with BPD be assessed and admitted to hospital for a short stay (up to 3 days) if they are at immediate risk of suicide [16, 107], approaches to hospital admissions among health practitioners varied considerably. Deans et al. [57] found that 89% of health practitioners reported that consumers *should be* managed in hospital; contrasting with 14.9% of health practitioners reporting that consumers with BPD *should not be* admitted to hospital. Other studies found that some health practitioners *do not view* hospitalization as an effective strategy [86, 110, 121]. For example, in James et al. [121], 64% of respondents reported that they agreed with the statement *‘patients with BPD should not be hospitalized’*. Health practitioners decision *not to hospitalize* consumers with BPD (unless at immediate risk of suicide) may stem from past experiences involving some consumers’ with BPD creating interpersonal conflict on the wards, such as splitting staff or negatively influencing other consumers [110]. Consequently, some consumers with BPD may fall through the service gaps (i.e., experience inconsistencies in the care provided and the recommendations for the delivery of evidence-based healthcare) [72], or disengage from treatment following negative experiences in their attempts to seek help from health services [115].

Other structural challenges and barriers in health systems impacting the provision of health services and crisis care for consumers with BPD include: limited funding allocated for BPD-specific resources such as access to longer term public and private health services [101]; lack of BPD-related health literacy for consumers and carers/families [1, 3, 4, 7, 34, 35, 61, 88]; concerns regarding insurance coverage and financing private therapeutic services [88]; the lack of evidence-based crisis interventions given there are currently no Randomized Control Trials (RCT) assessing the effectiveness of existing crisis interventions [35]; and, problems associated with discharge planning and continuity of care [8, 64, 107]. The structural challenges and barriers specific to carers/families included: lack of knowledge on BPD [35] and the skills to help the person they care for [2]; limited access to BPD-related health literacy, services, and supports to assist carers/families to cope and care for themselves as well as the person with BPD experiencing crises [1, 4, 62, 73, 89]; the lack of consultation with health practitioners regarding the care plans detailing the treatment and management of the person with BPD which limits carers/families ability to effectively care for the person with BPD [10].

#### Practitioner-consumer interactions

This sub-theme is centred around existing literature exploring the interpersonal dynamics and encounters that can arise during health practitioners, consumers with BPD, and carers/families interactions [3, 4, 7, 48, 49, 51, 65, 72, 85, 104, 109, 116, 119, 122]. Consumers with BPD commonly experience difficulties with interpersonal communication and conflicts which are reflected in their interpersonal relationships and experiences with health practitioners. Practitioners have been described in the literature as having an important mediating influence upon consumers with BPD [35]. For instance, qualitative studies found that interactions with health practitioners either relieved or increased the distress of consumers with BPD and their carers/families [8] with practitioners’ responses being perceived as helpful or discriminatory [72]. Consumers with BPD indicated that they felt socially isolated and rejected when health practitioners were perceived to be unapproachable [65], or abrasive. Indeed, Lawn et al. [3] found that more than half of consumer participants with BPD (53%, n = 60) recalled being treated disrespectfully by health practitioners. Consumers with BPD also indicated that they needed to stand up for themselves when interacting with health practitioners [62].

Similarly, carers/families of consumers with BPD reported being treated disrespectfully [4] or made to feel as though they were to blame, or responsible for, the presentation of the person with BPD [35]. Carers/families also recalled experiencing stigma by association (i.e., stigma extended to family members based on their relationship with the person that they care for) when they engaged with some practitioners. These interpersonal conflicts may retraumatize consumers with BPD and their carers/families and catalyse a crisis [35, 115]. Despite these negative encounters, consumers with BPD reported that they valued their connection with health practitioners, and wanted to have positive working relationships with practitioners [115].

**Fig. 2:**
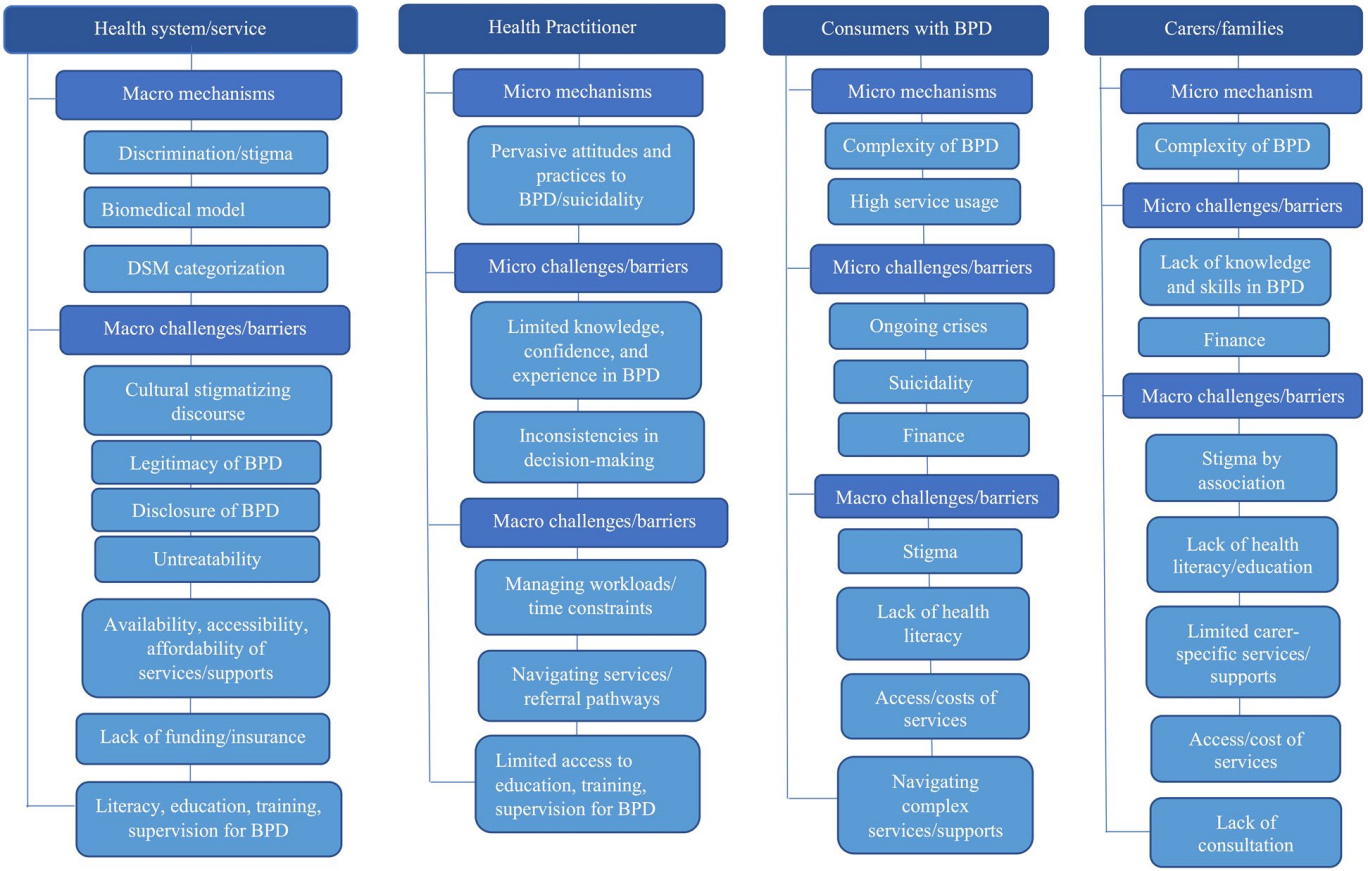
Macro- and micro level structural mechanisms, and barriers impacting healthcare for health practitioners, consumers with BPD, and carers/families

Extant literature suggests that health practitioners believe consumers with BPD are typically difficult to engage and interact with [114]. Psychiatric nurses perceived consumers with BPD as resistant to treatment, which made it stressful for the nurses to connect with these consumers and build rapport [86]. James et al. [121] found 75% of health practitioners considered consumers with BPD were moderately or very difficult; and 80% of participants believed that consumers with BPD were more difficult to engage than other consumers. Health practitioners also reported experiencing strong emotional reactions including, feeling uncomfortable, powerless, and professionally challenged when interacting with these consumers [84].Primary health practitioners [42] and allied health staff [113] also reported being concerned about their ability to effectively manage countertransference and practitioner-patient boundaries when working with consumers diagnosed with BPD. These negative reactions and encounters can create major barriers [86] to the development of effective communication and practitioner-patient relationships [33, 49], as well as with carers/families. Similar to consumers, health practitioners also reported that they wanted to improve their relationships with consumers with BPD and carers/families [76]. Figure 2 presents the various macro- and micro-level structural mechanisms, challenges, and barriers impacting the provision of healthcare for BPD. Additional file 3 outlines the structural factors influencing BPD-related structural stigma in healthcare systems across the relevant populations (i.e., health practitioners, consumers with BPD, and carers/families).

## Discussion

This scoping review systematically mapped and synthesized a narrative summary of international literature examining the impact of structural stigma on the provision of healthcare for people with BPD and their carers/families. The results presented here confirm that BPD remains a highly stigmatized and controversial mental illness that engenders inconsistent and often inadequate responses in health systems [1–4, 34, 35, 49, 52, 65, 68, 73]. Consumers with BPD and their carers/families face many sources of adversities when seeking help from health services [1–4, 10, 65, 73]. These adversities stem from several macro- and micro-level factors that contribute to the shortcomings in health services and care for BPD [34, 35, 42, 47, 90, 108, 114, 115, 118, 125]. The prominent structural mechanisms, challenges, and barriers impacting the delivery of responsive health services and care for BPD comprised: discrimination and stigma towards consumers with BPD and their carers/families [19, 34, 35, 49, 72, 106, 122, 126]; the inadequacy of the biomedical model of care in meeting the complex needs of BPD [114, 118]; the DSM grouping for BPD [12, 118]; and, the limited BPD-related resources including support services and education [3, 4]. These structural considerations have had a profound impact on the capacity of health systems to deliver responsive services and care to this population.

Several key themes and sub-themes emerged from the data highlighting the immense effects of structural stigma on consumers with BPD and their carers/families who endeavour to access health services and supports. Commonly held myths concerning the legitimacy of the BPD diagnosis, its disclosure to consumers, and the discourse of untreatability are interlinked, and combine to perpetuate the stigmatizing culture, attitudes, and practices evident within many health systems [3, 4, 35, 72, 106, 114, 118, 120]. Debates continue to question the validity of BPD as a mental health disorder—sometimes framing its symptomatology as an immoral discourse (e.g., acts of self-harming behaviour are an attempt to gain attention), rather than a diagnosis positioned within a illness discourse [35, 114]. This implies that consumers with BPD are not truly ill or deserving of healthcare and thus justify the refusal of treatment. Contrastingly, the evidence that health practitioners used to categorize consumers as *not being ill* (such as presenting with suicidal behaviour) could also be used to justify these consumers’ legitimate illness and need for treatment [114]. Chronic suicidality among consumers with BPD could instead be interpreted as a cry for help and a signal that these consumers are *not receiving* effective treatment to assist their recovery, despite their ongoing attempts to seek help [112]. The notion that receiving healthcare is contingent on particular definitions associated with being unwell [114] violates peoples’ most fundamental right to access healthcare [16]. These findings suggest a need to recognize and respond to the serious nature of crisis presentations in health systems given consumers with BPD are a patient group at high-risk of suicide [28, 114].

The extent of the structural stigma associated with BPD has had a substantial influence on health practitioners’ diagnostic and disclosure practices [19, 114]. The complexity of BPD [12, 19], and the associated structural problems (i.e., lack of available treatment options, stigmatizing culture and practices, inadequate training of health professionals) have resulted in some health practitioners withholding a BPD diagnosis from their patients [19, 108, 114]; perceiving that giving this diagnosis would do more harm than good [115]. In contrast, this assumption has been refuted by other health practitioners [19, 123] and consumers with BPD who reported that receiving a diagnosis of BPD gave them relief and was a positive step towards understanding BPD, its associated symptoms, and accessing appropriate treatment [115].

Findings relating to health practitioners’ pervasive stigmatizing attitudes and practices to BPD underscore the structural problems that are woven into the cultural fabric of health systems [34]. Studies indicate that some health practitioners may hold prejudice towards BPD, limiting their ability to explore and understand the underlying causes of self-harming behaviour among consumers with BPD [123]. For consumers with BPD, their experiences of repeated crises are complex and multidimensional, and may compound their level of distress, as well as the distress experienced by their carers/families and health practitioners [45]. Evidence suggests that health practitioners also find these experiences challenging, and may feel powerless in the context of self-harm, and overwhelmed by the chaotic and conflictual interactions partly due to the lack of knowledge, strategies, and skills to manage crisis situations [34]. It is likely that health practitioners’ stigmatizing attitudes and externalizing practices of blame and refusal of care, arise from being overwhelmed and uncertain about how to manage the situation [34]. Sansone and Sansone [90] argued that health practitioners’ negative reactions to consumers with BPD may simply reflect a normal human response to the complexity and pathological nature of these consumers. The extent to which the stigma of BPD is largely situated within health systems is an indication that the problems and solutions lie in the culture and delivery of health services and care, and not the BPD diagnosis [60], and requires concerted effort to address the impact of structural stigma on healthcare for this population [92]. These findings highlight the importance of investing in BPD-related education, training, and supervision to assist health practitioners to better support consumers with BPD and their carers/families when they engage with health services during crises [45, 54, 77, 89, 106, 109, 110].

This review has implications for health service design and the delivery of responsive treatment and crisis interventions to better support consumers with BPD and their carers/families. The literature identifies several recommendations for addressing BPD-related structural stigma and improving health service delivery including the utilization of whole-of-system approaches to addressing structural stigma at both the macro- and micro-levels of healthcare institutions [34, 42, 47, 48, 52, 110, 113, 116]. This involves implementing coordinated and targeted approaches that address the structural factors associated with BPD-related stigma in health systems including: the cultural norms, policies, and practices [16, 47, 107]; implementing psychosocial models of care for BPD [60] underpinned by person-centred and compassionate approaches [35, 77]; and, increasing funding for BPD-related resources [41] which include professional development for staff.

Clear recommendations have detailed the need for health services to not only treat the physical ailments associated with self-harm, but also, the underlying emotional distress that is associated with self-harm and suicidality among consumers with BPD [60]. These recommendations require holistic approaches to care delivery including increased access to longer-term specialized therapeutic services [42, 49]. In addition, the absence of RCT confirming the effectiveness of existing crisis interventions for BPD [79] makes clinical decisions regarding evidence-based treatment and management of BPD challenging. This warrants urgent instigation of high-quality research to investigate the efficacy of crisis interventions for BPD [35]. At a system level, structural stigma concerning BPD in healthcare systems must be addressed. Research investigating the various multi-levels and multifaceted components of BPD-related stigma is needed to explicate how changes in each of these structural factors interact and operate (either separately or together) to impact the health and wellbeing of this population. This may include testing the specific effects of the structural mechanisms identified in this review in relation to their impact on health service access, service delivery, and health outcomes of people with BPD and their carers/families [80].

## Limitations

There are several limitations to this review. Studies included in this review may have an inherent bias in terms of their research question (e.g., attracting participants who have had bad experiences with the health system). It is important to acknowledge that despite the structural barriers and associated stigma discussed in this review, these findings may not apply across all health systems, particularly health and community-based services providing high quality BPD-related services and care to community. Another limitation involved BPD being the primary diagnosis explored within the context of structural stigma and its impact on healthcare therefore, data pertaining to experiences of structural stigma relating to other mental health disorders (including personality disorders) were not captured. Also, publications were limited to English only, and some relevant publications may have been missed due to the exclusion of full-text publications that were unable to be accessed free of charge, or older publications that may not have been available for download. Furthermore, there is a lack of high-quality RCT which restricted the findings of this review such that inference of causality regarding the health impacts of structural stigma [79] could not be applied, nor generalized to the broader population [9]. This includes the lack of effectiveness studies available to support the use of evidence-based crisis interventions for BPD [79].

## Conclusion

Consumers with BPD and their carers/families often experience recurring crises and frequently seek help from healthcare services. Structural stigma specific to BPD remains pervasive in health systems, reflected by many macro- and micro-level factors that are embedded in institutional policies, cultural norms, and practices. Key structural mechanisms impacting the delivery of adequate services for BPD were identified in the literature. These included BPD-related stigma and discrimination, and the dominance of the biomedically-driven approaches to healthcare. Implications for future practice and research were discussed, along with recommendations for addressing BPD-related stigma in healthcare systems including the need for holistic system-wide changes to service delivery that are underpinned by psychosocial and person-centred approaches.

## Supplementary Information

**Additional file 1: **Search strategy for various databases.

**Additional file 2: **PRISMA 2009 checklist.

**Additional file 3: **Data extraction of the findings on Borderline Personality Disorder (BPD) related structural stigma in healthcare systems.

## Data Availability

Not applicable.

## Abbreviations

BPD: Borderline Personality Disorder
CINAHL: Cumulative Index to Nursing and Allied Health Literature
JBI: Joanna Briggs Institute
MMAT: Mixed Methods Appraisal Tool 2018 version
PCC: Population-Concept-Context framework
PRESS: Peer Review of Electronic Search Strategies
PRISMA-ScR: Preferred Reporting Items for Systematic Reviews and Meta-analyses; Extension for Scoping Reviews: Checklist and Explanation
RCT: Randomized Controlled Trials
