# Supplementary material for: Structural stigma and its impact on healthcare for borderline personality disorder: a scoping review
Source: Int J Ment Health Syst. 2022 Sep 29;16:48. doi: 10.1186/s13033-022-00558-3 (PMC9520817; doi:10.1186/s13033-022-00558-3)
Supplement: Supplementary file 3 — Additional file 3: Data extraction of the findings on Borderline Personality Disorder (BPD) related structural stigma in healthcare systems. [file 13033_2022_558_MOESM3_ESM.docx]

**Additional file 2: Data extraction of the findings on Borderline Personality Disorder (BPD) related structural stigma in healthcare systems**

| **Author,**  **Year, Country** | **Population Type/Setting** | **Key findings on Structural Policies and Procedures**  (For example, professional development) | **Key findings on Clinical Practice**  (For example, knowledge, attitude, behaviour) |
| --- | --- | --- | --- |
| Citations focused on health practitioners | | | |
| Bodner et al. 2011,  IL | Psychiatrists  Psychologists  Nurses/ Psychiatric services | - Lack of formal training in BPD-related treatments i.e., Dialectical Behaviour Therapy - Psychologists found to be more familiar with BPD-related therapies than psychiatrists and nurses | - Lack of education and training in BPD-related therapies - Stigmatizing attitudes and behaviours - All practitioner types (psychiatrists, psychologists, nurses) found to have stigmatizing attitudes toward BPD, including: - cognitive attitudes - required treatment, suicidal tendencies, antagonistic judgment - emotional attitudes - negative emotions, experienced-difficulties in treatment, empathy - Professional experience - seniority associated with more empathy and less negative emotions towards patients with BPD. |
| Bodner et al. 2015,  IL | Psychiatrists, Psychologists,  Social Workers Nurses/  Psychiatric hospitals | - Need for education and training to assist health practitioners to improve skills | - Stigmatizing attitudes and behaviours - cognitive attitudes - negative emotion attitudes - Nurses and psychiatrists exhibited more negative attitudes/less empathy toward patients with BPD than psychologists and social workers - BPD diagnosis perceived more negatively by all professions than other diagnoses - Interest in education and training/improving skills. |
| Clarke et al. 2015,  UK | Multi-disciplinary teams/ Inpatient setting | - Limited BPD-related anti-stigma education and training | - Stigmatizing beliefs and attitudes of health practitioners towards BPD - Lack of knowledge in BPD - measures included: mental health locus of origin, empathetic concern, perspective taking. |
| Commons Treloar et al. 2008,  AUS | Medical and health practitioners/ Emergency  medicine, Mental health services | - Need for BPD-related education and training | - Stigmatizing attitudes of emergency medicine or mental health practitioners towards BPD and deliberate self-harm - Predictors of attitudes to self-harm were type of health service, that is, emergency medicine or mental health; years of experience, and specific training in personality disorders. |
| Commons Treloar et al. 2009a,  AUS | Medical and health practitioners/ Emergency  medicine, Mental health services | - Inadequacies of the health system/service responses in addressing BPD patient needs - Strategies/approaches needed to improve service provision - Lack of resources, including limited staff - Lack of regular education, training, and supervision to assist shifts in cultural norms/stigmatizing practices and improve therapeutic relationships/practices - Using care plans and case managed by a small number of clinicians to avoid team conflict/tensions regarding treatment and management of people with BPD | - Interpersonal difficulties including uncomfortable negative health practitioner attitudes/service responses to people with BPD/self-harming behaviours - Discriminatory and stigmatizing practices identified as: withholding or refusing treatment/services, biased clinical assessments to people with diagnosis of BPD. |
| Commons Treloar et al. 2009b,  AUS | Registered  health practitioners/ Healthcare services | - BPD-related education and training - Need for increased education and training on therapeutic approaches to assisting health practitioners to support people with BPD | - Negative attitudes towards of BPD. |
| Day et al. 2018,  AUS | Mental health nurses/  Public health service | - Need for more effective treatments that meet the needs of people with BPD | - Attitudes and skills in working with people with BPD and deliberate self-harm - Negative attitudes and experiences of working with this population. |
| Deans et al. 2006,  AUS | Registered psychiatric nurses/ Psychiatric inpatient and community services | - Conflicting views of health practitioners in relation to the treatment and management of BPD, despite the release of the Clinical Guidelines for the Management of BPD including, hospitalization, medication, and generalist vs specialist care | - Negative attitudes towards people with BPD and suicidality - A striking finding is that less than half of the respondents (44%) reported that they knew how to care for people with BPD. |
| Dickens et al. 2016,  UK | Mental health nurses | - Need for BPD-related education and training (on aetiology and Dialectical Behaviour Therapy), and the translation of knowledge from training into improved practice and clinically significant outcomes such as improved therapeutic relationships, service user satisfaction, or reductions in self-harming behaviour - Need to identify the specific components of effective education and training for BPD | - Cognitive (beliefs about aetiology, mental health locus of origin) and affective (perspective taking, willingness to disclose, and desire for social distance, attitudes) and behaviour towards BPD and deliberate self-harm. |
| Dickens et al. 2019,  UK | Mental health nurses | - System-wide changes in service provision for BPD needed - Need for BPD-related education and training - Lack of BPD-related resources to support staff - Use of expert by experience identified as an effective component of education and training for BPD | - Staff knowledge, attitudes, practice, and confidence in working with people with BPD. |
| Hauck et al. 2013,  USA | Psychiatric nurses/ Psychiatric hospitals | - Need for further BPD-related education and training to enhance positive attitudes towards BPD and deliberate self-harm. - Low scores in nurses' years of service and self-reported need for further education and training correlated with more negative attitudes which may compromise quality of care | - Negative attitudes towards BPD and deliberate self-harm. |
| James et al. 2007,  IL | Psychiatric nurses/ Psychiatric services | - Lack of services identified as the most important factor contributing to the inadequate care and the development of a specialist service is reported as the most important resource to improve care - 81% of respondents believe that the care people with BPD receive is inadequate | - Psychiatric nurses’ knowledge, experience, and attitudes towards clients with BPD. |
| Keuroghlian et al. 2006, USA | Medical practitioners  Psychologists, Counsellors, Nurses /  Medical Services | - BPD-related education and training needed to enhance health practitioners’ confidence and competence in treating this population | - Health practitioners’ beliefs, attitudes, and perceived competence in treating people with BPD - Fewer years of clinical experience was related to a greater increase in feeling professionally competent to care for patients with BPD. |
| Knaak et al. 2015,  CA | Psychiatrists,  Psychologists,  Social workers,  Counsellors, Nurses,  Director,  Managers/ Health services | - Need for anti-stigma interventions, education, and training to address structural stigma in healthcare settings | - Healthcare provider negative attitudes (attitudes towards mental illness, disclosure/help-seeking) and behavioural intentions (preference for social distance) towards persons with BPD. |
| Koehne et al. 2013,  AUS | Medical staff,  Psychologists, Nurses,  Occupational Therapists/ Child and Adolescent Mental Health Services | - Lack of transparency in communications and diagnosis disclosure, i.e., doctors, nurses and allied health clinicians resist/subvert a diagnosis of BPD among adolescents | - Inconsistency in practices across clinical teams regarding the use of social and discursive strategies used by clinicians, including: - cultural norms impacting team rules which discouraged diagnostic disclosure - the lexical strategy of hedging when using the diagnosis - the prohibition and utility of informal ‘borderline talk’ among clinicians - reframing the diagnosis with young people. |
| Ma et al. 2009,  TW | Mental health nurses/ Psychiatric Health Centre | N/A | - Negative attitudes and experiences impacted on practitioner/patient interactions and decisions regarding care delivery - Need for a team approach to the delivery of care of people with BPD as the practice of routine or individualized care strategies, with or without adequate support from other team members, produces satisfactory or unsatisfactory outcomes. |
| Markham 2003a,  UK | Mental health nurses/  Mental health  inpatient facilities | N/A | - Stigmatizing attitudes and perceptions among mental health nurses towards BPD on social distance, dangerousness, optimism, and personal experience measures - Staff rated negative experiences of working with people with BPD higher than other mental illnesses. |
| Markham et al. 2003b,  UK | Mental health nurses/  Mental health  inpatient facilities | N/A | - Staff reported greater negative attitudes and perceptions towards people with BPD compared to other mental illnesses on empathy/sympathy, optimism for positive change, control over behaviour, and personal experience measures. |
| Masland et al. 2018,  USA | Mental health practitioners, researchers/ Medical centres | - Limited brief education and training opportunities to equip health practitioners with the tools and skills to effectively work with people with BPD | - Stigmatizing beliefs, attitudes, and practices toward BPD. |
| McGrath et al. 2012,  IE | Registered psychiatric nurses | - Suboptimal levels of care provided to people with BPD in healthcare systems/services | - Stigmatizing attitudes and regarding the challenges and difficulties associated with delivering a 'good level of care' to people with BPD among psychiatric nurses - Avoidance/refusal to provide services/care to people with BPD. |
| Millar 2012,  SC | Psychologists | - Working in contrast to the system relates to concerns/inconsistencies regarding the diagnosis of BPD and its impact on access to health services/ supports - Need for increased BPD education, training and supervision, and opportunities to liaise/consult with psychiatrists | - Negative feelings, attitudes, and experiences of working with people with BPD were found within eight suboptimal themes, including: ‘negative perceptions of the client’, ‘undesirable feelings in the psychologist’, ‘positive perceptions of the client’, ‘desirable feelings in the psychologist’, ‘awareness of negativity’, ‘trying to make sense of the chaos’, ‘working in contrast to the system’ and ‘improving our role’. |
| Nehls 2000,  USA | Health practitioners/ Community Mental Health Centre | - Discriminatory practices in limiting access to health services/supports by Case Managers as a way of setting boundaries in response to excessive demands on the case manager-client relationship/service | - Case Managers denying clients with BPD denied activities in community-based mental health programs - Limiting access to services/programs a barrier to building and maintaining effective therapeutic relationships. |
| Pigot et al. 2019,  AUS | Health practitioners | - Need for further BPD-related education and training for mental health service practitioners to address barriers services for BPD - Increase resource allocation to support crisis care interventions in healthcare services. | - Addressing staff knowledge, attitudes, and skills for BPD. - Sites where managers impeded change by not providing adequate resources was a barrier to successful implementation of the stepped care intervention - Lack of engagement by medical staff was another barrier to successful implementation. |
| Shaikh et al. 2017,  USA | Health practitioners/ Emergency department | - Challenges in diagnosis, crisis care interventions, and negative attitudes among health practitioners in emergency departments contribute to the structural stigma experienced by people with BPD - Education and training targeting specific health practitioners including ambulance staff is needed to improve positive attitudes, compassion, and patience toward people with BPD | - Negative attitudes and behaviours of health practitioners treating people with BPD in emergency services. |
| Sitsti 2016,  USA | Psychiatrists | - Some psychiatrists have reported withholding and/or not documenting a patient's diagnosis of BPD due to stigma or uncertainty of the diagnosis. - Clinical education and training needed to address these issues | - Psychiatrics decision to not disclose a diagnosis of BPD to protect their patient/s from structural stigma. |
| Stroud et al. 2013,  UK | Registered Community Mental Health Nurses/ Community Mental Health Team | - Service-related constraints/issues impacted the knowledge, attitudes and approach health practitioners used with clients, including: - the need for education, training, and supervision for BPD - limited resources and service demands such as high caseloads and documentation processes, and, - risk management and litigation | - Limited knowledge and understanding of BPD among health practitioners - Negative attitudes and interactions with people with BPD. |
| Sulzer 2015,  USA | Psychiatrists, Psychologists,  Social Workers/  Inpatient and out-patient settings | - Stigmatizing experiences of patients with BPD being routinely labelled “difficult,” and subsequently routed out of care through a variety of direct and indirect means. - This process creates a functional form of de-medicalization where the actual diagnosis of BPD and the treatment component of medicalization is harder to secure for patients | - Stigmatizing and discriminatory practices including the denial of treatment to people with BPD are based on stereotypical assumptions including the untreatable nature of the disorder, and the discourse of the disorder being untreatable and difficult to treat. |
| Sulzer 2016a,  USA | Psychiatrists, Psychologists,  Social Workers | - Most health practitioners did not disclose a diagnosis of BPD despite patients' preferences for being informed of their diagnosis and its associated stigma. - Education and training needed to address this issue | - Poor communication practices regarding the disclosure of a diagnosis of BPD. Many patients learn about their diagnosis from another source other than the practitioner that made the diagnosis, resulting in some patients leaving treatment prematurely. |
| Sulzer 2016b,  USA | Health Practitioners | - Stigma as a consequence of the bio-medical mix-match in the treatment and management of BPD - Longer-term therapeutic interventions required to support the complex needs of people with BPD - Resource limitations associated with insurance costs/reluctance of insurance companies to cover treatments | - Inconsistency in health practitioners use of the biomedical model of care as the dominant model, with some practitioners using a mixture of approaches to support patient care - Health practitioners' attitudes and behaviours regarding misunderstandings/myths associated with BPD including the disorder being untreatable. |
| Warrender 2015,  UK | Nurses/ Acute mental health services | - Need for education, training, and supervision in therapeutic approaches to address stigma and service-level challenges relating to BPD | - Nursing staffs' perspectives and practice in the delivery of services and care to people with BPD - Training in therapeutic approaches helped to empower staff to change their attitudes and practice empathetic approaches to care for people with BPD. |
| Wlodarczyk et al 2018,  AUS | General Practitioners (GP)/ GP Partners Australia | - Structural barriers obstructing GP in the provision of care to people with BPD - Challenges facing GP providing care to patients with BPDs impact care including, challenges surrounding diagnosis, comorbidities and clinical complexity, difficulties with patient behaviour and the practitioner–patient relationship; and identifying and navigating systems for support is needed - Education, training, and support for GP is needed to improve health services for BPD | - Health service pathways and quality care for people with borderline personality are dependent of GP capacity to identify and understand the disorder. |
| Woollaston et al. 2008,  UK | Nurses | - Negative experiences of nurses working with patients with BPD impact the quality of services and care provided to people with BPD | - Negative perspectives, attitudes, and behaviours of nurses towards BPD and suicidality. |
| Citations focused on people with BPD | | | |
| Borschmann et al. 2014,  UK | People with BPD/  Community | The need for people with borderline personality to have:   - joint crisis plans that are individually tailored to their needs - more autonomy in decision-making | - Unhelpful interactions with mental health professionals during crises - Unhelpful actions by mental health professionals including - Need for people with BPD to be treated with dignity and respect and the receive emotional and practical support from health practitioners. |
| Fallon 2003,  UK | People with BPD/ Psychiatric services | - Accessibility of health services and approachability of health practitioners viewed as important by people with BPD - Frequent use of health services during times of crises associated with suicidality - Recognition that health care service responses had improved overtime, with the implementation of the care programme approach CPA - Relationships with staff viewed as valuable resources by this population - A complex healthcare system to navigate, lacking continuity of care at times, and a mixture of negative and positive experiences that impact rapport building and effective long-term relationships with health practitioners | - Experience negative attitudes and experiences of accessing health services, however people with BPD still find their contact with psychiatric services valuable - Relationships with others were important in containing distress despite having trust issues. |
| Horn et al. 2007,  UK | People with BPD | - Practitioner-level power and control over knowledge - Impact of diagnosis label on service provision stem from discrimination, stigma, and judgements of limited BPD - Lack of fit between health system/services and BPD | - Withholding knowledge of diagnosis, providing limited BPD-related information, and practitioners as experts - Services rejected or withheld following disclosure of diagnosis. |
| Lawn et al. 2015a,  AUS | People with BPD | - Stigmatizing and discriminatory service responses towards BPD in public and private health systems - Challenges accessing BPD-related health and community services, supports, and other resources | - Stigmatizing attitudes and practices including, refusal of hospitalization/treatments during crises - Inconsistency in the level of support offered by health practitioners to people with BPD. |
| Morris 2014,  UK | People with BPD | - Significant gaps in health services, and disjointed and unreliable care provision in the health system | Key themes relating to participants experiences of mental health services included:   - the diagnostic process influenced how service users felt about, and their level of understanding of, BPD - experiences of non-caring care including poor communication, reluctance of health practitioners to work with them based on the diagnosis and a lack of support - Need for the development of positive trusting relationships with staff resulted in amplified distress through increasing feelings of ‘‘emptiness’’ and alienation. |
| Nehls 1999,  USA | People with BPD/ Psychiatric, outpatient, and community services | - Need to address prejudice associated with BPD in healthcare systems - Limited access to health services and care for BPD | - Lived experiences of stigmatizing and discriminative attitudes, judgements, and behaviours from health practitioners in relation to the diagnosis of BPD and self-harm. |
| Perseius et al 2005,  SE | People with BPD | - Lack of continuity in healthcare services and care delivery | - Experiences of not being understood and disrespectful attitudes towards people with BPD. |
| Proctor et al. 2020,  AUS | People with BPD | - Stigma in healthcare systems associated with a lack of knowledge, understanding and training in BPD - Need for adherence to clinical practice guidelines in the treatment and management of BPD including transparency and communication of the diagnosis | - Limited access to responsive health services and supports for BPD/crisis care intervention - Individually tailored care plans need to be developed and managed in collaboration with consumers and carers/families involvement. |
| Rogers 2012,  UK | People with BPD | - Lack of knowledge and understanding of the BPD diagnosis impacts upon the delivery of care to people with BPD - Lack of resources to support responsive services, supports and health literacy | - Challenges in the treatment of BPD stem from stigmatizing attitudes, and a lack of knowledge and understanding of the condition. |
| Stapleton et al. 2019,  UK | People with BPD/  Acute Psychiatric  inpatient wards | - Need for education and training on BPD to improve service provision and the experiences of care for people with this condition | - Negative attitudes and lack of knowledge of BPD affects service provision and care. for this population. |
| Vandyk et al. 2019,  CA | People with BPD/ Emergency departments | - Stigmatizing healthcare system/service experiences among people with BPD who attempt to access health services and care during crises | - Negative attitudes of health providers towards BPD who present to an emergency department in crisis - People with BPD engagement of coping skills and strategies to avoid attending emergency departments during a crisis. |
| Veysey 2014,  NZ | People with a BPD | - Stigmatizing and discriminatory healthcare systems and practices impact the quality of services as well as participants' experiences of both helpful and/or discriminatory practices | - Negative attitudes and behaviours associated with BPD and self-harm among health practitioners. |
| Citations focused on carers/families of people with BPD | | | |
| Acres et al. 2019,  AUS | Carers/  Emergency care | - Lack of adherence to clinical practice guidelines such as, the National Health and Medical Research Council guidelines 2012 - Limited education and training on BPD - Staying within the confines of the medical model limits the development of meaningful patient/practitioner interactions and avoids the challenges inherent in the complexity of diagnosis of BPD | - Lack of education and training on BPD - Lack of communication between health practitioner/carer - a barrier to treatment - Carer refused involvement in treatment/care planning - limits carers knowledge/information of condition, treatment, and continued support of person with BPD - Lack of knowledge/understanding of BPD - Stigma towards suicidality - limited access/refusal of medical treatment affects the therapeutic relationship/compromises trust. |
| Buteau et al. 2008 | Carers/families | - Challenges accessing health services/treatments - Challenges navigating the Mental Health Services - Lack of BPD-related information and resources - Financial burdens associated with expensive healthcare services/treatments | - Lack of engagement and support from health practitioners. - Health practitioners who were unapproachable triggered feelings of isolation among people with BPD - Negative attitudes of psychiatric hospital nursing staff resulted in patients feeling undeserving of inpatient care. |
| Dunne-Rogers et al. 2013,  UK | Carers/ Community Personality Disorder Service | - Ineffective carers assessments - Lack of financial support/financial assessment opportunities for BPD - Lack of supports and resources for carers | - Lack of support from health practitioners - Poor engagement, inclusion and collaboration in care plans and treatment for the person they care for – carers experiences of being ‘overlooked’. |
| Ekdahl et al. 2011,  SE | Carers | - Lack of BPD-related resources and information for carers - Lack of a holistic approach to BPD service provision | - Negative encounters with psychiatric and health services – feeling left out and abandoned as not included in care planning/treatment processes - Lack of trust with psychiatric care delivery/therapeutic relations with psychiatric practitioners. |
| Lawn et al. 2015b,  AUS | Carers | - Carers experience stigma and discrimination in their attempts to gain services and support from health services - Inadequate community support services for carers of people with BPD - Lack of BPD-related resources and information to support carers of people with BPD - Inadequate crisis care service responses | - Experiences of exclusion and discrimination when attempting to interact with health practitioners regarding the person they care for - Limited engagement, involvement, and consultation with carers during crisis planning, treatment and management of people with BPD - Inconsistencies in care responses to crisis and suicidality exists among different types of health practitioners. |
| Citations across health practitioners, people with BPD, carers/families | | | |
| Carrotte et al. 2019,  AUS | People with BPD,  Carers | - Macro and micro level challenges exist within health service organizations regarding the complexity of navigating the system/ services - Mechanisms of structural stigma include, costs, service access, clinician factors and client factors - Experiences were often not aligned with a recovery model of mental health - Health access - lack of services/supports including, specialist services | - Lack of knowledge/understanding of BPD among carers/families and health practitioners, fosters misinformation - Poor communication and reluctance of health to diagnose BPD - Insufficient information to support carers - Lack of education and training of health practitioners - Unethical and unprofessional behaviours of health practitioners. |
| Lohman 2017,  USA | People with BPD, carers/families/ Hospitals, Resource Centre | - Limited access to responsive mental health services in response to the stigmatization of BPD - Insufficient resources including, family services, crisis intervention, and health literacy to support consumers and carers affected by BPD - Financial cost of treatment as barriers to accessing health services | - Negative attitudes and practices surrounding BPD and suicidality - Lack of services/supports for carers of people with BPD to help alleviate stress relating to their caring role. |
| Ng 2016,  AUS | Health practitioners, people with BPD,  carers/families | - Stigma associated with the diagnostic label hindered trust formation and a consumer’s ability to fully engage | - Need for improvements in practitioner-patient relationships to support continued engagement in treatment and recovery - Carers want to be involved in collaborating on care plans and treatment for the person they are caring for. |
| Ring et al. 2019,  AUS | People with BPD,  Mental health professionals’ | - The impact of stigma associated with BPD at the interface of mental health care on the diagnosis, treatment, and management of this condition - Limited resources and health literacy for BPD - Need for education and training to increase empathy and decrease stigma associated with for BPD | - Preconceived stigmatizing attitudes and behaviours towards people with BPD limits access to responsive health services and care. |
| Warrender et al. 2020,  UK | Health Practitioners, people with BPD, carer/families/ various healthcare settings | - Challenges in accessing responsive health services and crisis care interventions including the refusal of services in psychiatric services and emergency departments - Limited resources result in a lack of choice of services and support for BPD - Need for education, training, and supervision for BPD to improve health service provision and care for this population | - Stigmatizing and discriminatory attitudes and practices among health practitioners, including the refusal of services to people with BPD in crisis - People with BPD preferences for care include access to therapeutic relationships that offer emotional and practical support - Carers want health practitioners to involve them in the care planning and treatment of the person they care for to assist them to understand the condition and offer continual support. |
| Clinical Practice Guidelines | | | |
| National Health and Medical Research Council 2012,  AUS | Clinical practice guidelines for the treatment and management of BPDs | - National standards of quality services and care for BPD - The recognition that BPD is a legitimate mental illness requiring clinical treatment and management - Non-adherence to clinical practice guidelines on the effective management of people with BPD | - Stigmatizing and discriminatory practices including, health practitioners refusing to treat, or withholding services from, people with BPD. |
| The British Psychological  Society and The Royal College of Psychiatrists, 2009 | Clinical practice guidelines for the treatment of BPDs | - Advises on the National standards for improving quality services, diminishing unacceptable variations in the provision and quality of patient-centred care for BPD | - Public health intervention guidance in the delivery of clinical practice and care for BPD to eliminate stigmatizing and discriminatory practices of people with borderline personality and their carers/ families. |
